# Light-Mediated Kinetic Control Reveals the Temporal Effect of the Raf/MEK/ERK Pathway in PC12 Cell Neurite Outgrowth

**DOI:** 10.1371/journal.pone.0092917

**Published:** 2014-03-25

**Authors:** Kai Zhang, Liting Duan, Qunxiang Ong, Ziliang Lin, Pooja Mahendra Varman, Kijung Sung, Bianxiao Cui

**Affiliations:** 1 Department of Chemistry, Stanford University, Stanford, California, United States of America; 2 Department of Applied Physics, Stanford University, Stanford, California, United States of America; 3 Biophysics Program, Stanford University, Stanford, California, United States of America; Hungarian Academy of Sciences, Hungary

## Abstract

It has been proposed that differential activation kinetics allows cells to use a common set of signaling pathways to specify distinct cellular outcomes. For example, nerve growth factor (NGF) and epidermal growth factor (EGF) induce different activation kinetics of the Raf/MEK/ERK signaling pathway and result in differentiation and proliferation, respectively. However, a direct and quantitative linkage between the temporal profile of Raf/MEK/ERK activation and the cellular outputs has not been established due to a lack of means to precisely perturb its signaling kinetics. Here, we construct a light-gated protein-protein interaction system to regulate the activation pattern of the Raf/MEK/ERK signaling pathway. Light-induced activation of the Raf/MEK/ERK cascade leads to significant neurite outgrowth in rat PC12 pheochromocytoma cell lines in the absence of growth factors. Compared with NGF stimulation, light stimulation induces longer but fewer neurites. Intermittent on/off illumination reveals that cells achieve maximum neurite outgrowth if the off-time duration per cycle is shorter than 45 min. Overall, light-mediated kinetic control enables precise dissection of the temporal dimension within the intracellular signal transduction network.

## Introduction

Through a delicate network of interacting proteins, intracellular signaling pathways transmit various extracellular signals into the intracellular environment to regulate gene expression and determine cell fate. A single extracellular stimulus often simultaneously activates multiple signaling pathways. Conversely, the same signaling pathway can be activated by diverse cellular stimuli [1]. To ensure proper conversion of specific environmental inputs to distinct cellular outputs, cells exploit temporal control of signaling pathways so that a common set of these pathways can yield diverse biological responses [2], [3], [4], [5], [6].

A good example is the Raf/MEK/ERK signaling pathway, which plays a vital role in cell proliferation, differentiation, and apoptosis [7], [8], [9], [10]. Extensive studies of the Raf/MEK/ERK pathway have suggested that its functional outcome depends on its activation kinetics [11], [12]. For instance, although EGF and NGF trigger similar sets of signaling pathways in PC12 cells including Raf/MEK/ERK [7], [8], PI3K/AKT [13], and PLCγ pathways [14], [15], EGF induces cell proliferation while NGF induces cell differentiation accompanied by cell cycle arrest [3], [16], [17], [18], [19]. The difference in the temporal profile of ERK activation, i.e. transient activation by EGF versus sustained activation by NGF, is believed to be primarily responsible for the distinct cell fates [3], [20], [21], [22], [23], [24], [25], [26]. In other studies, persistent activation of ERK is shown to be involved in glutamate-induced toxicity in neurons [27], [28]. Therefore, it has been suggested that whether the Raf/MEK/ERK signaling pathway induces cell proliferation, differentiation, or death is determined by the temporal duration of pathway activation [29].

Despite the obvious importance of the temporal dimension of the Raf/MEK/ERK pathway, there are very limited means to probe this dimension with high accuracy. Previous attempts to manipulate activation kinetics have involved the use of different growth factors, overexpression of heterologous receptors, or long-term drug application, most of which lead to changes in multiple signaling pathways, and none of which allows precise temporal control [22], [26], [30], . The challenge arises partly from intrinsic downstream feedback mechanisms that modulate the activation states of signaling components. For instance, it has been shown that EGF-induced ERK activation diminishes after 15 min despite the continuous presence of EGF due to rapid degradation of EGF receptors and other feedback mechanisms [33]. An added level of complexity is the temporal pattern of pathway activation. In two studies by Ji et al. and Chung et al., the same growth factor elicited different cellular responses depending on the time-dependent application of the growth factor [25], [34]. As a result, a quantitative understanding of the Raf/MEK/ERK signaling pathway is lacking, and it is unclear whether differential kinetics of one signaling pathway is sufficient to mediate the different effects of NGF and EGF on PC12 cells.

Several different methods have been developed to achieve time control of intracellular signal transduction. A rapamycin-induced FKBP-FRB heterodimerization system has been developed to control various protein activities and cellular processes [35], [36], [37], [38]. This approach is generally applicable to many systems but lacks precise spatial and temporal control. This limitation was overcome by the use of a photocaged rapamycin analogue [39] or a direct photocaged protein kinase [40]. However, these approaches are not reversible and the UV light used in photo-uncaging could potentially induce phototoxicity. In the last few years, several genetically encoded photo-switchable protein pairs, including light, oxygen, or voltage domain (LOV) [41], [42], [43], [44], cryptochrome (CRY2) [45], [46], and phytochrome [47], [48], [49] have been developed. In particular, the cryptochrome CRY2-CIBN dimerization system is generally applicable, does not require any small molecule cofactor, and can be induced with very low intensity of light. A recent study achieved light-controlled activation of the Raf/MEK/ERK pathway based on the dimerization/aggregation of cryptochrome under blue light stimulation [50]. However, this system is not designed for live cell imaging and it has yet to be demonstrated whether this system is compatible with tracking cell behavior for extended time periods.

In this paper, we construct a light-gated Raf/MEK/ERK activation and inactivation system based on the CRY2 and CIBN protein pair. We demonstrate that a low intensity of blue light allows precise temporal control of the Raf/MEK/ERK activity over several days. In the absence of growth factors, light-induced Raf/MEK/ERK activation stimulates significant neurite outgrowth in PC12 cells. Light stimulation induces longer neurites than can be achieved by NGF at saturating concentrations. However, NGF induces more neurites per cell than light does, likely due to light-induced selective activation of Raf/MEK/ERK pathway in contrast to NGF-induced activation of multiple downstream pathways. Intermittent on/off light stimulation reveals a 45-min time threshold in neurite outgrowth – when the off-time per cycle is less than or equal to 45 min, the length of neurite remains the same as that stimulated by continuous light. When the off-time is greater than 45 min, the final neurite length decreases with increased off-time.

## Results and Discussion

### Design Scheme

**Figure 1:**
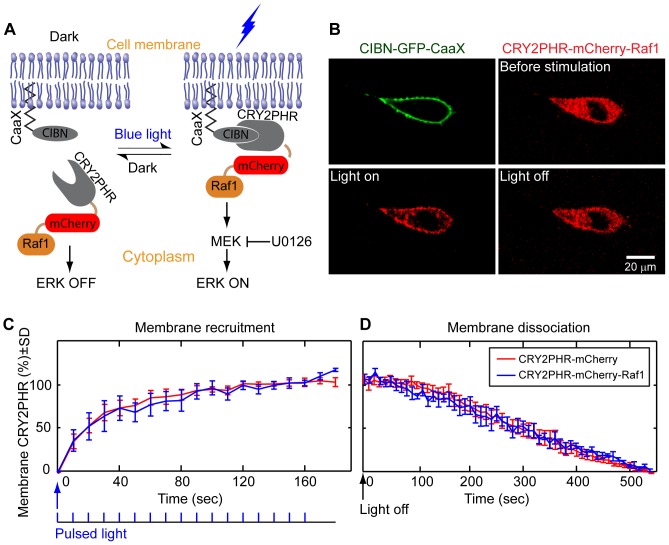
Schematic and characterization of the light-activated Raf/MEK/ERK signaling pathway. (A) The CIBN domain was anchored to the plasma membrane via a CaaX motif. Upon light stimulation, CIBN-CRY2PHR interaction should recruit cytoplasmic CRY2PHR-mCherry-Raf1 to the plasma membrane. Membrane recruitment of Raf1 should subsequently activate MEK and ERK. In the absence of light, spontaneous dissociation of CIBN-CRY2PHR should return Raf1 to the cytoplasm and inactivate ERK. (B) The CIBN-GFP-CaaX was clearly located at the plasma membrane as indicated by the green fluorescence. Before light stimulation, CRY2PHR-mCherry-Raf1 diffused homogenously in the cytoplasm, as expected for a cytosolic protein. Blue light rapidly recruited CRY2PHR-mCherry-Raf1 to the plasma membrane. When the blue light was switched off, CRY2PHR-mCherry-Raf1 dissociated from the plasma membrane and returned to the cytoplasm. Membrane recruitment (C) and dissociation (D) kinetics of CRY2PHR-mCherry and CRY2PHR-mCherry-Raf1 are quantified. The membrane/cytoplasm intensity ratio was plotted over time and normalized between 0 and 1 (the data and the error bars represent 8 and 4 independent experiments for CRY2PHR-mCherry and CRY2PHR-mCherry-Raf1, respectively). On average, 3 pulses of blue light (80 ms pulse duration, 10 s pulse interval) were sufficient to recruit 50% of CRY2-mCherry-Raf1 from the cytoplasm to the plasma membrane while the dissociation was much slower with a half-life time of 5 min. Data are presented as mean ± SD.

The Raf protein is a kinase with a C-terminal kinase domain and a negative regulatory domain close to the N-terminus. Whereas inactive Raf is cytosolic, it is activated upon membrane recruitment by activated Ras. It was previously reported that targeting Raf1 (also known as c-Raf) to the plasma membrane via a farnesylation signal was sufficient to activate Raf1 independent of its activator Ras [51], [52]. Inspired by this study, we hypothesized that precise and reversible control of the Raf/MEK/ERK pathway could be achieved if we were able to use light to recruit Raf1 to the plasma membrane. For this purpose, we chose a light-gated protein interaction system consisting of the truncated form of cryptochrome 2 (CRY2PHR) and its binding partner CIBN from *Arabidopsis thaliana* [45], [53], which was shown to bind within seconds when exposed to blue light and dissociate within a few minutes when the blue light was shut off. We used two fusion proteins to achieve light-induced membrane recruitment of Raf1. The first protein is CIBN fused with a green fluorescence protein (GFP) and a C-terminal K-Ras CaaX domain, which targets the CIBN domain to the plasma membrane. The second protein is CRY2PHR-mCherry fused with Raf1 [45]. When blue light is turned on, membrane-anchored CIBN should bind to CRY2PHR and thus recruit CRY2PHR-mCherry-Raf1 to the plasma membrane, where Raf1 should be phosphorylated and activated and subsequently turn on downstream kinases in the Raf/MEK/ERK pathway. When blue light is turned off, CYR2PHR-mCherry-Raf1 should dissociate from CIBN and return to the cytoplasm, inactivating the Raf/MEK/ERK pathway (Fig. 1A).

### Light induces Raf1 membrane recruitment via CRY2PHR-CIBN interaction

We first demonstrated that blue light was able to recruit the CRY2PHR-mCherry-Raf1 protein from the cytoplasm to the CIBN-decorated plasma membrane. NIH 3T3 fibroblasts were co-transfected with CIBN-GFP-CaaX and CRY2PHR-mCherry-Raf1 plasmids. As confirmed by the GFP fluorescence in Fig. 1B (top left image), the CIBN domain was anchored to the plasma membrane. Before blue light illumination, CRY2PHR-mCherry-Raf1 diffused homogenously in the cytoplasm, as expected for a cytosolic protein (Fig. 1B, top right image). We used short pulses of blue light as the stimulating light source (pulse duration 80 ms per scan, pulse interval 10 s, 2 MW/cm^2^ peak intensity on a standard Nikon A1 confocal microscope). As shown in Fig. 1B (bottom left image), blue light rapidly recruited CRY2PHR-mCherry-Raf1 to the plasma membrane. For the same cell, switching off blue light released the membrane bound CRY2PHR-mCherry-Raf1 to the cytoplasm (Fig. 1B, bottom right image, also see Movie S1). The cycle of membrane-recruitment/release could be repeated in the same cell for multiple times (>5) until photobleaching hindered further detection.

We quantified the association and dissociation kinetics by measuring the change in membrane/cytoplasm fluorescence intensity ratio, normalized between 0 and 1. Averaging over four experiments, the kinetics showed that 3 pulses of blue light were sufficient to recruit 50% of CRY2-mCherry-Raf1 from the cytoplasm to the plasma membrane (Fig. 1C). We used pulsed blue light to avoid significant photobleaching during the kinetic measurement. When the blue light was turned off, CRY2PHR-mCherry-Raf1 dissociated from the membrane to the cytoplasm with a half-life time of about 5 min (Fig. 1D). The association and dissociation kinetics agrees with previous report [45]. We detected no significant difference in the CRY2PHR-CIBN association and dissociation kinetics between CRY2PHR-mCherry-Raf1 and CRY2PHR-mCherry, indicating that the Raf1 domain did not perturb the interaction between CIBN and CRY2PHR (Fig. 1C–D).

### Light-induced membrane recruitment of Raf1 leads to ERK activation

**Figure 2:**
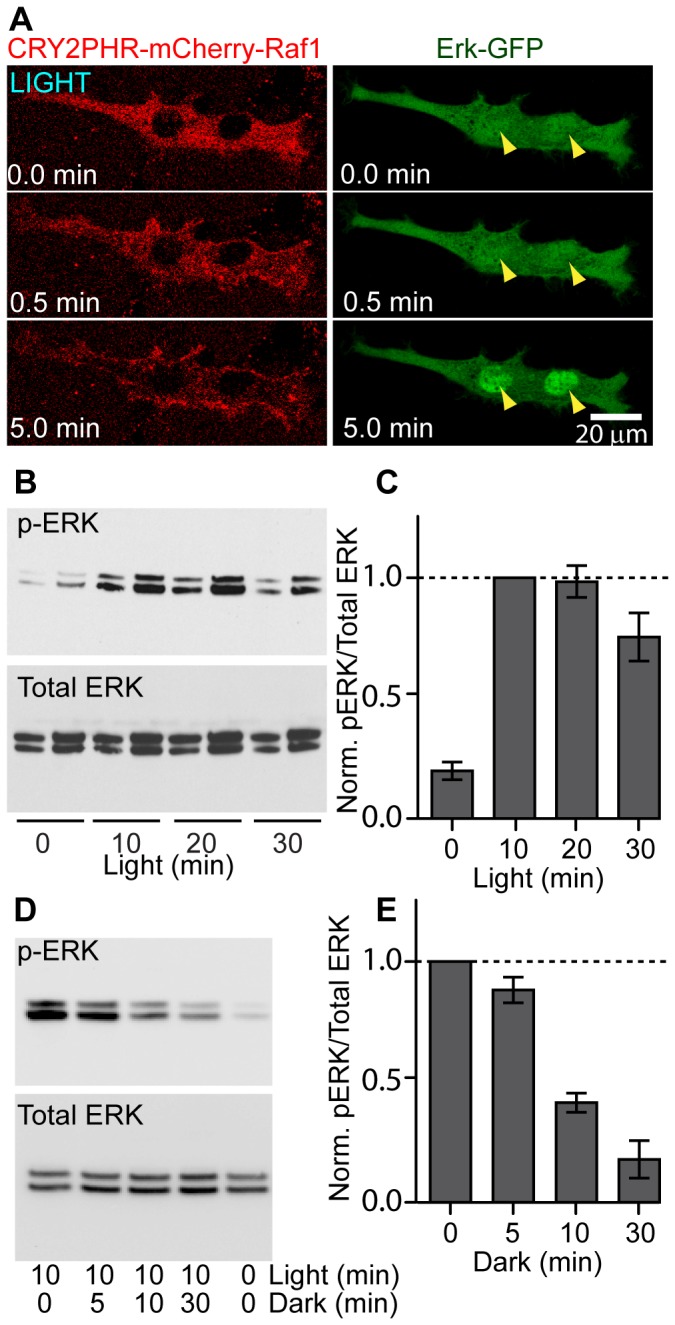
Light-induced Raf/MEK/ERK activation assayed by ERK2-GFP nuclear translocation and ERK phosphorylation. (A) NIH 3T3 fibroblasts were triply transfected with CIBN-CaaX, CRY2PHR-mCherry-Raf1, and ERK2-GFP. Under blue light stimulation, CRY2PHR-mCherry-Raf1 was recruited from the cytoplasm to the plasma membrane, which was accompanied by a concurrent translocation of ERK2-GFP from the cytoplasm to the nucleus. Nuclear accumulation of ERK2-GFP was clearly visible after 5 min (yellow arrow heads). (B) Western blot analysis of phosphorylated ERK (pERK at Thr202 and Tyr204) upon light stimulation. Two gel lanes were used for each time point, loaded with 10- and 20-μL protein samples respectively. The level of pERK was shown to increase significantly after 10, 20 or 30 min of light stimulation. (C) Normalized intensities of each band in B with respect to the intensity of the band of 10-min light-stimulation. Intensities of the 20-uL bands were used for bar-graph analysis. The bar graph was presented with mean ± standard error (s.e.m.), averaged over three independent sets of experiments. (D) Western blot analysis of dephosphorylation of pERK upon switching off the light after 10-min light stimulation. The level of pERK quickly decayed as the dark incubation time increased. (E) Normalized intensities of each band in D with respect to the intensity of the band of 0-min dark time. The bar graph is presented with mean ± standard error (s.e.m.), averaged over two independent sets of experiments.

ERK translocates from the cytoplasm to the nucleus upon activation [54], [55], [56]. Therefore, we used the ERK nuclear translocation assay to test whether light-induced Raf1 membrane recruitment could activate the Raf/MEK/ERK signaling pathway, in the absence of growth factor stimulation. NIH 3T3 cells were triply transfected with CIBN-CaaX, CRY2PHR-mCherry-Raf1, and ERK2-GFP. In order to avoid blue-light stimulation when visualizing GFP, the mCherry channel (∼560 nm excitation) was used to locate the transfected cells. Before blue light stimulation, both CRY2PHR-mCherry-Raf1 and ERK2-GFP were mostly in the cytoplasm (Fig. 2A and Movie S2). Blue light rapidly recruited Raf1 from the cytoplasm to the plasma membrane. Concurrent with Raf1 membrane recruitment, ERK2-GFP translocated from the cytoplasm to the nucleus (Fig. 2A right panels and Movie S2).

We further confirmed the light-induced ERK activation by Western blot analysis of phosphorylated ERK (pERK, Thr202 and Tyr204). NIH 3T3 cells co-transfected with CIBN-CaaX and CRY2PHR-YFP-Raf1 were incubated in low-serum medium (0.1% fetal bovine serum) for 24 h to reduce the pERK background before light stimulation. Each cell culture was stimulated by blue light for 0, 10, 20, or 30 min, followed by immediate cell lysis and Western blot analysis. As shown in Fig. 2B, the amount of pERK increased significantly after 10 min of light stimulation, which remained around the same level with 20-min stimulation and decayed slightly with 30-min stimulation (Fig. 2B). The experiment was repeated three times and the pERK level was normalized against that of 10-min light illumination (Fig. 2C). To confirm that pERK can be inactivated upon switching off the light, four co-transfected cultures were first exposed to 10 min of blue light stimulation and then were incubated without light for 0, 5, 10, and 30 min before cell lysis. After blue light was shut off, the pERK level monotonically decayed as the dark incubation time increased (Fig. 2D), reaching ∼10% peak level after 30 min without light (Fig. 2E). We also confirmed with Western blot that 30-min blue light stimulation did not induce phosphorylation of stress-activated protein kinase/c-Jun N-terminal kinase (SAPK/JNK) (Fig. S1).

### Light allows reversible kinetic control of ERK activation

**Figure 3:**
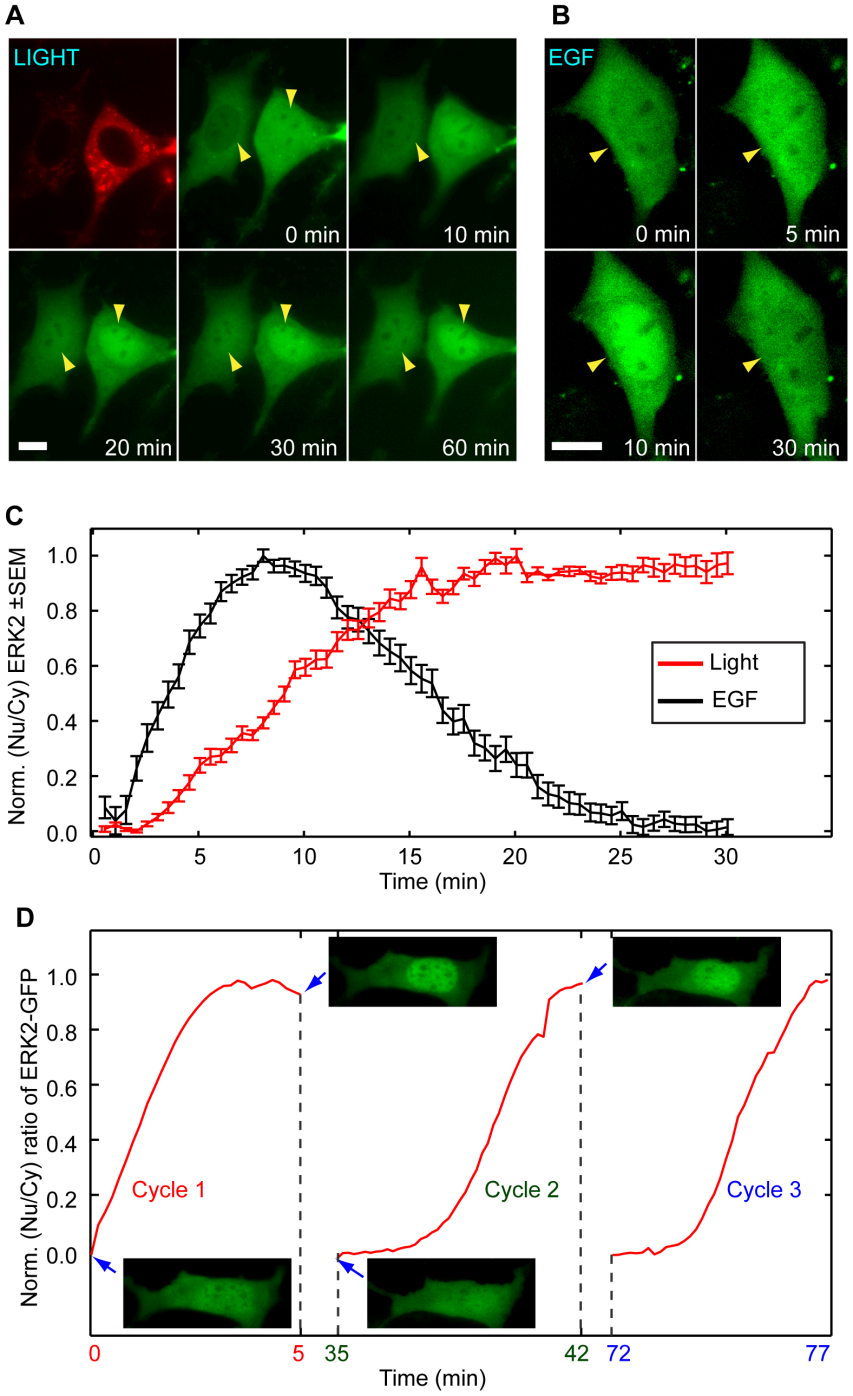
Kinetic control of ERK2-GFP activation by light. (A) For light-induced ERK nuclear translocation, ERK2-GFP stayed within the nucleus as long as the blue light stimulation was maintained (Movie S3). No cytoplasmic retreatment of the nuclear ERK2-GFP was observed even after 60 min of light stimulation (yellow arrows). (B) On the other hand, EGF-induced ERK2-GFP nuclear localization was transient. Nuclear ERK2-GFP peaked around 10 min and decayed to pre-treatment level by 30 min (yellow arrow heads) despite the continuous presence of EGF (white arrows). (C) The normalized nuclear/cytoplasmic ERK2-GFP ratio, averaged over 10 cells, clearly showed distinctive kinetics of ERK activation under light and EGF stimulation. EGF stimulation induced a peak followed by a monotonic decay to the background, while light stimulation induced a sustained level as long as the light was on. (D) Reversible control of ERK activation by light. The same transfected cell was repeatedly subjected to light and dark cycles. For each light illumination period, ERK2-GFP translocated from the cytosol to the nucleus. After each dark period of 30 min, the ERK activity decreased to the base level. Snapshots of the cell at different time points were shown. Scale bar in A and B: 10 μm.

Next, we showed that light could be used to precisely control the activation kinetics of the Raf/MEK/ERK pathway. Using the ERK nuclear translocation assay, we found that ERK2-GFP stayed within the nucleus as long as the light stimulation was maintained (Fig. 3A and Movie S3). No cytoplasmic retreatment of the nuclear ERK2-GFP was observed even after 60 min of light stimulation. On the other hand, EGF treatment induced a transient nuclear translocation of ERK2-GFP that peaked around 10 min. The nuclear ERK2-GFP decayed to the pre-treatment level after 30 min despite the continuous presence of EGF (Fig. 3B and Movie S4). The distinctive kinetics of ERK activation induced by light and EGF was clearly resolved by the normalized ratio of nuclear/cytoplasmic ERK2-GFP fluorescence intensity. As shown in Fig. 3C, light stimulation induced a sustained ERK nuclear localization as long as the light was on (red curve), while EGF stimulation induced a peak followed by a monotonic decay to the background (black curve). The onset kinetics of light-induced ERK translocation could be tuned by modulating the frequency of light pulses: a light train with 30-s interval resulted in the maximal nuclear ERK at 15 min (Fig. S2 red curve), which was reduced to 5 min when stimulated by a light train with 2-s interval (Fig. S2 blue curve). In addition, the light-induced ERK activity was reversible and repeatable (Movie S5). Figure 3D shows 3 cycles of ERK activation/inactivation of the same cell under light stimulation. 30 minutes of dark incubation returned the ERK activity to the base level.

### Light-induced activation of the Raf/MEK/ERK pathway is sufficient to stimulate PC12 cell differentiation in the absence of NGF

**Figure 4:**
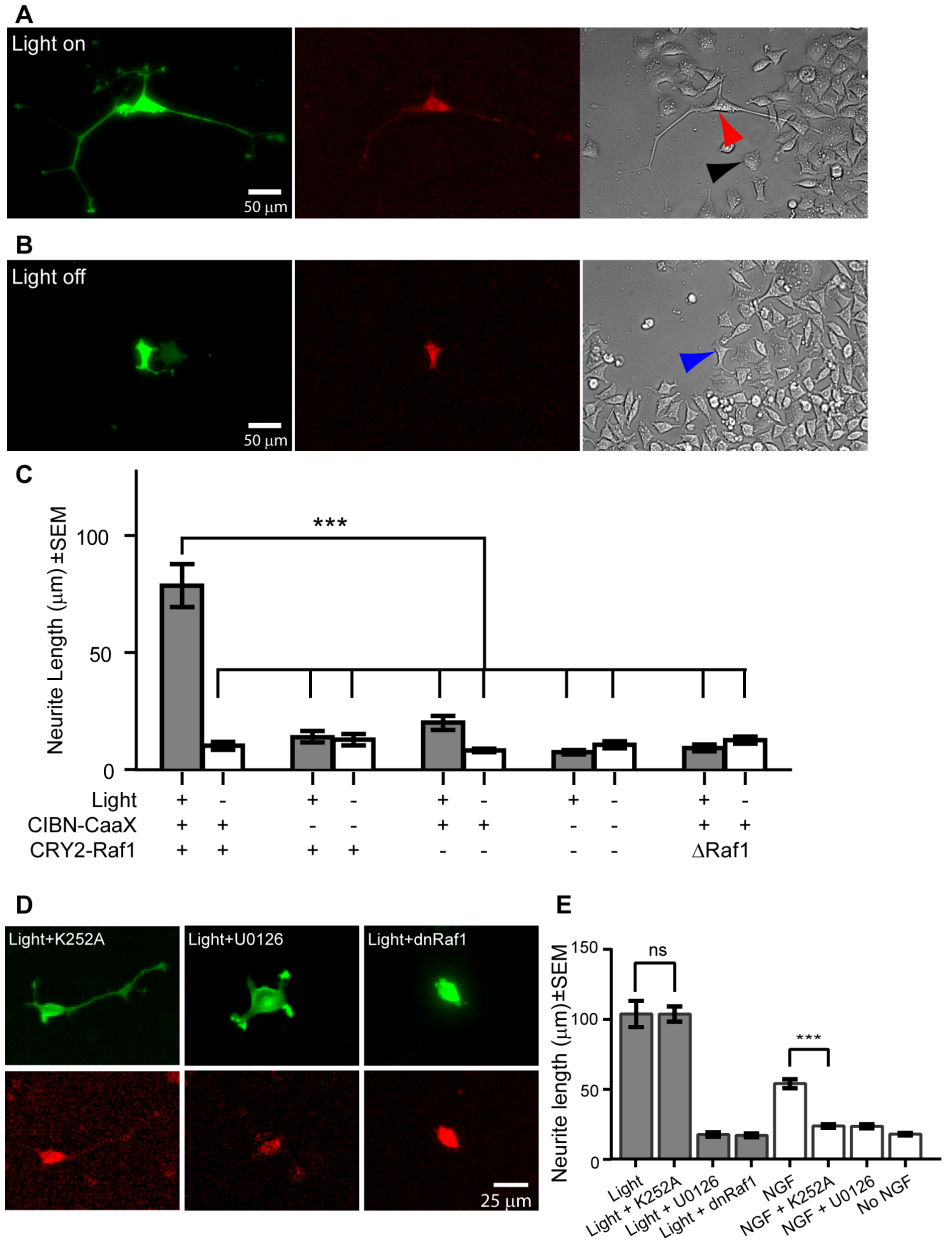
Light-induced Raf/MEK/ERK activation leads to significant neurite outgrowth in PC12 cells. (A) Three days of light stimulation induced significant neurite outgrowth in cells co-transfected with CIBN-GFP-CaaX and CRY2PHR-mCherry-Raf1 (red arrow head). Non-transfected cells in the same culture showed no marked neurite outgrowth (black arrow head). (B) When kept in the dark, cells co-transfected with both plasmids failed to grow long neurites after the same period of time, which is similar to that of non-transfected cells in the same culture (blue arrow head). (C) Quantitative analysis of neurite outgrowth in PC12 cells under various conditions. Under continuous light stimulation at 0.08 mW/cm^2^ (gray bars), co-transfected cells grew neurites significantly longer than those of singly transfected cells, non-transfected cells, or co-transfected cells in the dark. Removal of the Raf1 domain from CRY2PHR-mCherry-Raf1 abrogated the ability of co-transfected cells to grow out long neurites. ***p<0.001. The number of cells in each condition is n = 51 (light+co-transfection), n = 45 (dark+co-transfection), n = 23 (light+CRY2PHR-mCh-Raf1 single transfection), n = 18 (dark+CRY2PHR-mCh-Raf1 single transfection), n = 42 (light+CIBN-GFP-CaaX single transfection), n = 37 (dark+CIBN-GFP-CaaX single transfection), n = 30 (light+no transfection), n = 30 (dark+no transfection), n = 23 (light+co-transfection(ΔRaf1)), n = 23 (dark+co-transfection ΔRaf1). Data are presented with mean ± standard error (s.e.m.). Each condition was repeated for two sets of independent experiments. (D) Light-induced neurite outgrowth was blocked by U0126, a MEK inhibitor, but not by K252A, a TrkA inhibitor. (E) Quantifications of NGF and light induced neurite outgrowth with or without inhibitors. ***p<0.001. The number of cells for each condition is n = 55 (light), n = 117 (light + K252A), n = 18 (light + U0126), n = 69 (light + dnRaf1), n = 64 (NGF), n = 107 (NGF + K252A), n = 79 (NGF + U0126), n = 144 (No NGF). The bar graph is presented with mean ± standard error (s.e.m.). Each condition was repeated for at least three independent sets of experiments.

As NGF activates several signaling pathways, we set out to examine whether light-induced Raf/MEK/ERK activation alone could steer PC12 cells toward differentiation in the absence of NGF. The length of the longest neurite of each cell was measured to quantify the extent of PC12 cell differentiation [57]. A home-built blue LED array was used as the stimulating light source. During the experiment, the LED array was placed below the 12-well culture dish inside a 5% CO_2_ incubator. Twenty-four hours after co-transfection with CIBN-GFP-CaaX and CRY2PHR-mCherry-Raf1, PC12 cells were subjected to 0.08 mW/cm^2^ continuous light illumination in a low-serum culture medium (1.5% horse serum and 0.25% fetal bovine serum). After three days of light stimulation, we observed that the transfected cells grew out neurites as long as 300 μm (Fig. 4A, red arrow head), while non-transfected cells in the same culture rarely showed neurites longer than 20 μm (Fig. 4A, black arrow head). In the absence of light stimulation, cells co-transfected with both plasmids failed to grow long neurites after the same period of time (Fig. 4B, blue arrow head). Under our experimental condition, a three-day continuous illumination did not induce visible phototoxicity in PC12 cells.

To confirm that light-induced neurite outgrowth is due to light-induced Raf/MEK/ERK activation, we quantified the neurite length at various conditions by measuring the longest neurite for each cell [57]. Figure 4C shows that the average neurite length for co-transfected cells under light stimulation (78±10 μm, n = 51) was significantly longer than that of cells co-transfected with both plasmids but kept in dark (10±2 μm, n = 45), or that of non-transfected cells under light stimulation (7±1 μm, n = 30). Singly transfected cells, either with CIBN-GFP-CaaX (20±3 μm, n = 42) or CRY2PHR-mCherry-Raf1 (12±2 μm, n = 23) alone, showed no marked neurite outgrowth with or without light stimulation. The removal of the Raf1 domain from CRY2PHR-mCherry-Raf1 abrogated the ability of co-transfected cells to grow out long neurites (neurite length 9±1 μm, n = 23) under blue light stimulation (Fig. 4C). Representative cell images at different conditions can be found in Fig. S3.

As a positive control, a constitutively active, membrane-bound Raf1 (Raf1-GFP-CaaX) was able to induce long neurite outgrowth without light stimulation (Fig. S4A). We also confirmed that overexpression of wild type Raf1 did not induce neurite outgrowth, as cells transfected with either by CRY2-Raf1-mCherry (Fig. S3B) alone or Raf1-GFP alone showed no detectable neurite outgrowth (Fig. S4B), consistent with previous reports that overexpression of full-length wild-type Raf1 did not induce PC12 cell differentiation [58].

To confirm that light-induced neurite outgrowth requires the Raf1 kinase activity, we replaced wtRaf1 in CRY2PHR-mCherry-Raf1 with a dominant negative Raf1 (denoted as dnRaf1) that has a kinase-dead mutation (K375M). While CRY2PHR-mCherry-dnRaf1still responded to blue-light induced membrane recruitment, it failed to induce neurite outgrowth in PC12 cells under blue light stimulation (Fig. 4D). We further confirmed that light-induced Raf/MEK/ERK activation did not activate the NGF receptor TrkA that is upstream of Raf1 in NGF-induced Raf/MEK/ERK activation. Light-stimulated neurite outgrowth was not affected by the presence of TrkA inhibitor K252A (Fig. 4D), while NGF-induced neurite outgrowth was completely blocked (Fig. S5). On the other hand, the MEK inhibitor U0126 blocked neurite outgrowth by either NGF or light stimulation (Fig. 4D–E). Similar to measurements under light stimulation, the neurite length under NGF stimulation was also measured exclusively in co-transfected cells (Fig. S5). Fluorescence images with large field of view can be found in Fig. S6.

### Light induces neurite outgrowth in an intensity dependent manner

**Figure 5:**
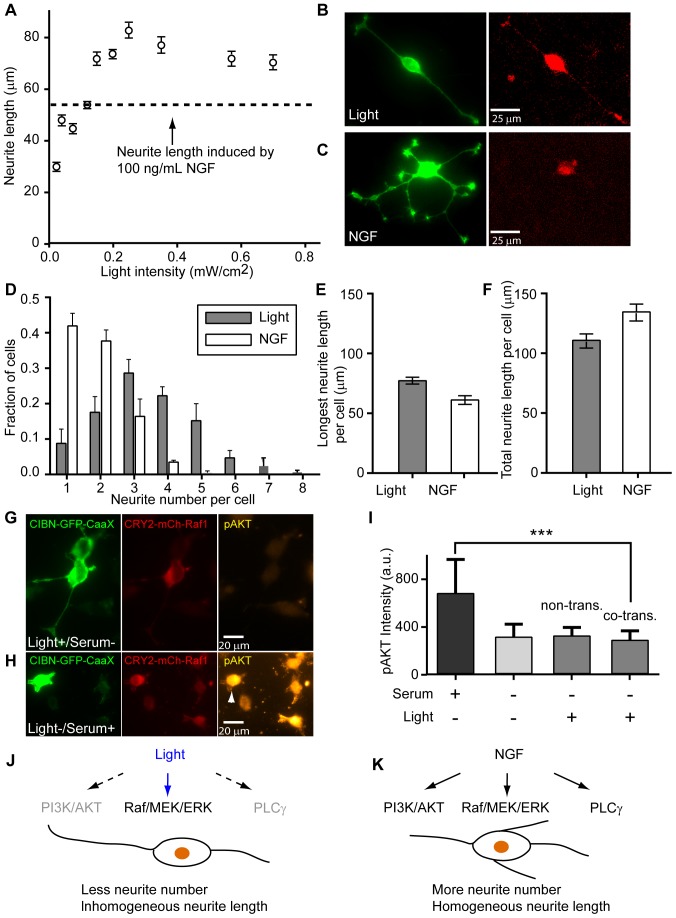
Light and NGF stimulation induce distinct morphologies in PC12 cells. (A) Light stimulation can induce longer neurites than NGF. The neurite length increased as the light intensity rose from 0.025 to 0.2 mW/cm^2^, and then leveled off at higher light intensity. NGF-induced neurite length is comparable to that induced by light at 0.08 mW/cm^2^. The number of cells in each condition is n = 72 (0.025 mW/cm^2^), n = 101 (0.04 mW/cm^2^), n = 93 (0.075 mW/cm^2^), n = 105 (0.12 mW/cm^2^), n = 121 (0.15 mW/cm^2^), n = 136 (0.20 mW/cm^2^), n = 102 (0.25 mW/cm^2^), n = 96 (0.35 mW/cm^2^), n = 106 (0.50 mW/cm^2^), n = 118 (0.70 mW/cm^2^), n = 64 (NGF). Each condition was repeated for two independent sets of experiments (Fig. S9). Data are presented with mean ± standard error (s.e.m.). Representative images of PC12 cell differentiation induced by light (B) versus induced by NGF (C) stimulation. Both light and NGF induced significant neurite outgrowth in PC12 cells, but NGF induced more complex neuritic structures. (D) The number of primary neurites induced by light versus that induced by NGF. PC12 cells grew out more primary neurites in response to NGF stimulation. (E) The average length of the longest neurite by light stimulation was longer than that stimulated by NGF. (F) NGF induced slightly longer total neurite length than light did. The number of cells is n = 171 (NGF), n = 167(light). Each condition was repeated for three independent sets of experiments. Data are presented with mean ± standard error (s.e.m.). Immunofluorescence staining for endogenous phosphorylated AKT (p-AKT at Ser473) in PC12 cells co-transfected with CIBN-GFP-CaaX and CRY2PHR-mCherry-Raf1. (G) Light-induced ERK activation in serum-deprived medium for 24 h stimulated neurite outgrowth but did not increase the p-AKT level. (H) Cells stimulated by 25% horse serum for 5 min showed significantly increased p-AKT level in the cytoplasm and at the plasma membrane (arrow head). All images in (G) and (H) were presented without adjusting the contrast or subtracting the background. (I) The level of p-AKT in serum-stimulated was significantly higher than that in serum-deprived or in light-stimulated PC12 cells, indicating that light-induced Raf1 membrane recruitment did not lead to AKT phosphorylation in 24 h. Overexpression of CIBN-GFP-CaaX and CRY2PHR-mCherry-Raf1 and light illumination did not induce AKT phosphorylation, as non-transfected and co-transfected cells showed similar p-AKT level. The bar graph was presented with mean ± standard deviation (SD). ***p<0.001. The number of cells in each condition is n = 54 (serum+, light+), n = 60 (serum-, light-), n = 55 (serum−, light+, non-transfected cells), and n = 42 (serum−, light+, co-transfected cells). (J–K) A schematic illustration showing that light and NGF induce different phenotype of cell differentiation in PC12 cells. Light-induced Raf/MEK/ERK activation results in longer but fewer neurites (J), while NGF induces a greater neurite number with homogeneous neurite length distribution (K). Such a difference suggests that the Raf/MEK/ERK signaling pathway is primarily responsible for neurite extension whereas other downstream pathways of NGF (PI3K/AKT and PLCγ etc.) play roles in neurite initiation.

Here, we investigated whether the length of the neurite depends on the intensity of the stimulating light using our custom built LED system. Figure 5A shows a power-dependent neurite length after 24 h of light stimulation with light intensity varying from 0.025 to 0.7 mW/cm^2^. The neurite length increased as the light intensity rose from 0.025 to 0.2 mW/cm^2^ and leveled off at higher intensity. By comparison, the neurite length induced by 100 ng/ml of NGF (shown as dashed line in Fig. 5A) was comparable to that induced by 0.08 mW/cm^2^ light and was shorter than that induced by 0.2 mW/cm^2^ of light. These results suggest that light can be used as a tool for an analog control of neurite outgrowth. For the following neurite outgrowth assays, we kept the light intensity at 0.2 mW/cm^2^, the minimum intensity that induces the maximum neurite outgrowth.

### Light and NGF induce distinct morphologies in PC12 cells

Although both light and NGF can induce significant neurite outgrowth in PC12 cells, the overall cell morphologies were different under two conditions. As shown in Fig. 5B, light stimulation caused cell to grow one or two long neurites per cell, while NGF stimulation often led to the growth of 4-5 neurites similar in length (Fig. 5C). Under 0.2 mW/cm^2^ of light stimulation, the average length of the longest neurite of each cell was longer than that induced by NGF at 100 ng/ml. On the other hand, the average number of primary neurite of each cell was 1.8 under light stimulation, fewer than the 2.9 neurites per cell induced by NGF (Fig. 5D). Such a difference in phenotype likely arises from the different downstream signaling pathways of light and NGF stimulation. NGF activates at least three signaling pathways (Raf/MEK/ERK, PI3K/AKT, and PLC<$>\raster(65%)="rg1"<$>) as discussed in the introduction, while light only activates the Raf/MEK/ERK signaling pathway. The neurite length analysis only measured the longest neurite for each cell (Fig. 5A, E). When we measured the total neurite length, we found that NGF and light stimulated similar total neurite outgrowth (Fig. 5F). Across experiments, we found that the neurite length and the number of primary neurite number showed little dependence on the CRY2PHR-mCherry-Raf1 expression level (Fig. S7A–B).

To confirm that light-induced ERK activation does not perturb other downstream pathways of NGF, we measured AKT phosphorylation in response to light stimulation. Using immunofluorescence staining, we probed the level of phosphorylated AKT (p-AKT at Ser473) in CIBN-GFP-CaaX/CRY2PHR-mCherry-Raf1 co-transfected PC12 cells under light stimulation (serum-free) or serum stimulation. The p-AKT levels in those cells were quantified by fluorescence intensity of an Alexa-fluor 647-conjugated secondary antibody (emission peak 690 nm, Fig. 5G–H). After light stimulation for 24 h in the serum-deprived medium, co-transfected cells showed no detectable AKT phosphorylation in either transfected or non-transfected cells (Fig. 5G) despite that light had induced long neurite outgrowth in transfected cells. Under serum stimulation, we detected significant p-AKT levels in transfected PC12 cells, as indicated by a bright fluorescence in the cytoplasm as well as at the plasma membrane (Fig. 5H arrow head). Averaging over 50 cells at each condition, we confirmed that light stimulation did not activate the AKT signaling pathway in co-transfected cells (Fig. 5I). For each fluorescent channel, images were acquired using the same exposure time for both serum and light-stimulated cells. All images are presented without contrast adjustment or background subtraction. Results from neurite number and length measurements and immunofluorescence staining experiments suggest that the Raf/MEK/ERK signaling pathway is primarily responsible for neurite extension, whereas other signaling pathways downstream of NGF stimulate the formation of multiple neurites (Fig. 5J–K).

### Continuous light stimulation is not required for maximum neurite outgrowth

**Figure 6:**
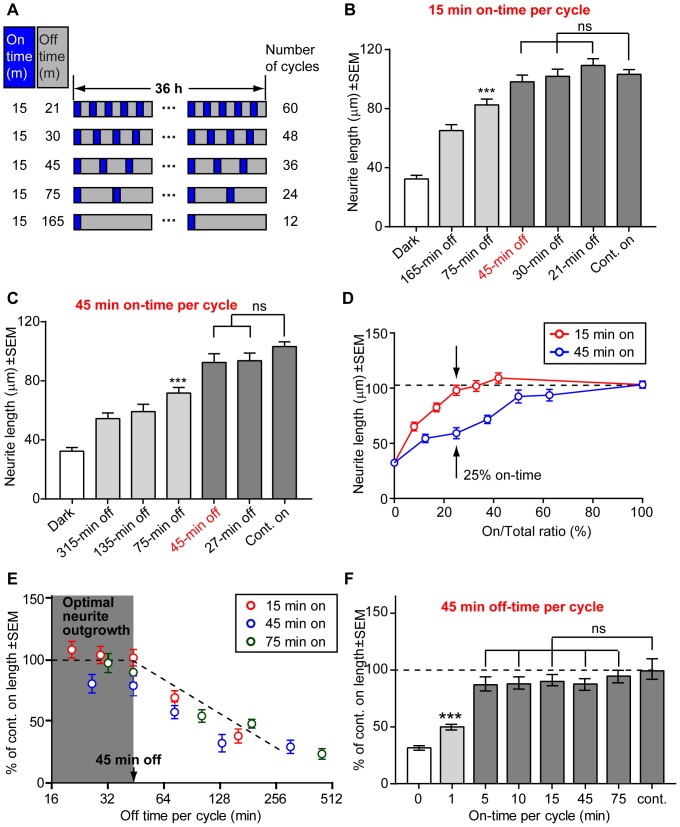
Effect of temporal control of the Raf/MEK/ERK pathway on PC12 neurite outgrowth. (A) Intermittent patterns of light stimulation. Cells were subjected to precisely controlled alternating cycles of light and dark periods for a total of 36 h. (B) The average neurite length induced by intermittent light stimulation with a 15-min on-time and various off-times at 21, 30, 45, 75 and 165 min. Intermittent light stimulation induced similar neurite length as continuous light stimulation when the off-time was less than or equal to 45 min. When the off-time exceeded 45 min, neurite length gradually decreased. ***p<0.001. (C) The average neurite length induced by intermittent light stimulation with a 45-min on-time and various off-times at 27, 45, 75, 135, 315 min. When the off-time was less than or equal to 45 min, similar neurite lengths were observed by intermittent and continuous light stimulations. ***p<0.001. (D) Neurite length versus the On-time/Total time percentage. The neurite length was not determined by the overall on-time percentage. Black arrows point to data for 15-min on/45-min off and 45-min on/135-min off, which had the same on-time percentage but the latter had much shorter neurite length. (E) Neurite length showed a 45-min off-time threshold, below which the neurite length was similar to continuous light stimulation (dark gray area). As the off-time increased beyond 45 min, the neurite length gradually decreased. Both the 45-min threshold and the gradual decay time were independent of the on-time. For all on/off intermittent light stimulation experiments, each condition was repeated for two independent sets of experiments (Fig. S10). For 15 min on-time, n = 76 (dark), n = 120 (165 min off), n = 121 (75 min off), n = 130 (45 min off), n = 139 (30 min off), n = 147 (21 min off), and n = 264 (Cont. on); for 45 min on-time n = 110 (dark), n = 121 (315 min off), n = 107 (135 min off), n = 153 (75 min off), n = 105 (45 min off), n = 126 (27 min off), and n = 330 (Cont. on); for 75 min on-time, n = 146 (dark), n = 109 (465 min off), n = 117 (195 min off), n = 127 (105 min off), n = 110 (45 min off), n = 115 (33 min off), n = 158 (Cont. on). (F) Neurite length versus on-time per cycle when the off-time was fixed at 45 min. Except for 1-min on-time stimulation, 5, 10, 15, 45, 75-min on-time all resulted in full-length neurite outgrowth, indicating that the neurite length was relatively independent of the on-time. ***p<0.001. The number of cells for each condition is n = 83 (dark), n = 84 (1 min on), n = 98 (5 min on), n = 56 (10 min on), n = 56 (15 min on), n = 105 (45 min on), n = 110 (75 min on), and n = 93 (Cont. on). Data are presented by mean ± standard error (s.e.m.).

Light allows precise and versatile control of Raf/MEK/ERK activation kinetics, which enables us to quantitatively measure how the activation kinetics determines neurite outgrowth in PC12 cells. We first investigated whether continuous light stimulation is required for maximum neurite outgrowth by using continuous or intermittent light stimulation. A LabVIEW computer program was used to create repetitive light stimulation patterns with variable light on and light off periods (Fig. 6A). The on-time and off-time durations for each LED were independently controlled so that 12 different light patterns could be measured simultaneously. The light power was kept constant at 0.2 mW/cm^2^. With continuous light stimulation, we observed that the average neurite length is ∼100 μm after 36-h light illumination. For intermittent light stimulation, we fixed the on-time at 15 min and varied the off-time at 21, 30, 45, 75 and 165 min. As shown in Fig. 6B, the neurite length induced by intermittent light stimulation was similar to that induced by continuous light stimulation when the off-time was less than or equal to 45 min. When the off-time was longer than 45 min, the neurite length induced by intermittent light stimulation was shorter and showed gradual decay as the off-time increased (Fig. 6B). Scatter plots of neurite lengths (Fig. S8A-C) show that both the average length and the distribution span decreased as the off-time increased beyond 45 min, indicating that longer off-time leads to an overall shortening of neurite length in all cell populations (cells with long and short neurites) instead of a decrease of cell populations with extremely long neurites.

### Light-induced neurite outgrowth exhibits an off-time threshold that is independent of the on-time duration

To investigate whether the 45-min off-time threshold depends on the on-time duration, we increased the on-time to 45 min and varied the off-time at 27, 45, 75, 135 and 315 min. Interestingly, we found the same off-time threshold of 45 min, i.e. intermittent on/off light stimulation was able to induce similar neurite outgrowth as continuous light stimulation when the off-time was equal to or less than 45 min (Fig. 6C). We noted that the neurite length was not determined by the overall on-time percentage. For example, 15-min on/45-min off gave much longer neurite length than 45-min on/135-min off despite that they had the same on-time percentage of 25% (black arrows, Fig. 6D). Given the same on-time percentage, higher frequency light stimulation generally led to longer neurites.

Fig. 6E shows results pooled from three sets of intermittent light stimulation experiments, each set consisting of a fixed on-time and five different off-times. All three data sets (on-time 15 min, 45 min, and 75 min) approximately fall on the same curve that shows a 45-min off-time threshold (Fig. 6E). In all cases, when the off-time was equal to or less than 45 min, neurite outgrowth was comparable to continuous light illumination. As the off-time increased beyond 45 min, the neurite length gradually decreased in all cases, indicating that cells slowly lost their memories of the Raf/MEK/ERK pathway activation during the light-on period.

To investigate whether there is a minimal on-time requirement, we set the off-time at 45 min and varied the on-time from 1 to 75 min. Figure 6F shows that 5, 10, 15, 45, 75-min on-time stimulations all resulted in comparable neurite outgrowth to that induced by continuous light stimulation, while 1-min on-time resulted in significantly shorter neurite length. Scatter plots of the data show again that both the average neurite length and the distribution span decreased for 1-min on-time data (Fig. S8D), indicating a shortened neurite length across all cells. It is likely that the Raf/MEK/ERK pathway was insufficiently activated upon 1-min light stimulation. These results suggest that the 45-min time threshold for light-induced maximum neurite outgrowth in PC12 cells is relatively independent of the duration of prior Raf/MEK/ERK activation.

## Discussion

In this report, we demonstrated precise and reversible control of the Raf/MEK/ERK signaling activity utilizing light-mediated CRY2PHR-CIBN interaction. Light-induced Raf/MEK/ERK activation was sufficient to induce neurite outgrowth in PC12 cells in the absence of growth factor stimulation. Light induced neurite outgrowth in an intensity-dependent manner, which is similar to the concentration-dependent manner of NGF-induced neurite outgrowth. However, it is intriguing that the activation of Raf/MEK/ERK pathway alone induced longer but fewer neurites compared with NGF. The results suggest that the Raf/MEK/ERK signaling pathway is primarily responsible for neurite extension whereas other downstream pathways of NGF binding play roles in neurite initiation.

The use of light as a control switch affords unique advantages in probing the temporal dimension of signaling pathways with high accuracy and versatility. Precise control of light on/off periods reveals a kinetic effect of light-stimulated neurite outgrowth in PC12 cells. In particular, we identified a 45-min off-time threshold. When the blue light was shut off, Raf1 dissociated from the plasma membrane within 10 minutes (Fig. 1D) while phosphorylated ERK decayed to less than 10% of the initial level in 30 min (Fig. 2C, Fig. 3D). Therefore, the observed 45-min threshold can be due to two reasons: (a) only very low level of pERK is needed to sustain full neurite outgrowth and/or (b) the signaling events downstream of ERK such as ERK-dependent activation of transcription factors can persist for much longer after the activity of Raf/MEK/ERK pathway decays [59], [60]. The time threshold is relatively independent of the prior duration of Raf/MEK/ERK activation.

Our result that single transfection of CRY2PHR-mCherry-Raf1 did not induce neurite outgrowth under light stimulation somewhat differs from a recent report where light-induced dimerization of CRY2PHR-Raf1 was sufficient to induce ERK activation [50]. One possible explanation is that the insertion of mCherry in our fusion protein prevented the dimerization of CRY2PHR or Raf1 due to steric hindrance. This is partially supported by their observation that the binding between cytosolic CIBN-Raf1 and cytosolic CRY2PHR-Raf1 did not induce p-ERK activity due to steric hindrance. In addition, we did not observe blue light-induced aggregation of CRY2PHR-mCherry-Raf1 under our experimental conditions as shown in Movie S1 and Movie S2. Since fluorescence proteins are widely used in live cell imaging, our design can be beneficial for applications where real-time visualization of protein location is preferred.

Our strategy of using light-gated protein interaction to probe the intracellular ERK pathway uses a special property of Raf1 kinase, i.e. auto-activation upon membrane recruitment. This property is shared by many other kinases such as PI3K and AKT, which have been shown to be activated upon membrane targeting [61]. Therefore, our strategy can be easily adapted to other signaling pathways.

## Materials and Methods

### Plasmids construction

CIBN-GFP-CaaX and CRY2PHR-mCherry were gifts from Dr. Chandra Tucker in University of Colorado Denver. ERK2-GFP was generously provided by Dr. XL Nan (now in Oregon Health and Science University). All other plasmids including CIBN-CaaX, CRY2PHR-mCherry-Raf1, and CRY2PHR-YFP-Raf1 were constructed using 2-step overlap extension PCR. The sequences of oligonucleotides used were summarized in Table S1; the PCR program was summarized in Table S2. In the first step, a PCR segment was made from the template containing the inserted gene using two primers that contain overlapping sequence with both the gene and the backbone. After the reaction, the PCR product was loaded into a 0.8% agarose gel (90 V, 30 min) and the PCR segment was extracted using the Gel extraction kit (Cat: 28704, Qiagen). The final concentration of the PCR segment was measured using a NanoDrop (Thermo Scientific). In the second step, the purified PCR segments were used as Mega primers for overlap extension PCR to be inserted into the backbone [62]. After the reaction, the product was subjected to an enzymatic digestion by 20 unit DpnI (R0176S, New England Biolabs) at 37°C for 2 h to remove the template. 2 μL of reaction mixture was used to transform 50 μL DH5α competent cells by heat shock. DNA was extracted from the resulted colonies and the sequence was confirmed by sequencing.

### Cell culture and transfection

NIH 3T3 fibroblasts (a generous gift from Prof. Matthew Scott in Stanford University) were used for characterizing membrane recruitment of CRY2PHR-mCherry-Raf1 and subsequent activation of ERK. Rat pheochromocytoma PC12 cells (a general gift from Prof. Tobias Meyer in Stanford University) were used for neurite outgrowth assays. For NIH 3T3 cells, we used DMEM medium supplemented with 10% fetal bovine serum (FBS). For PC12 cells, we used F12K medium supplemented with 15% horse serum and 2.5% FBS. All cell cultures were maintained a standard incubator at 37°C with 5% CO_2_.

For Western blot, 2.0×10^5^ NIH 3T3 fibroblast cells were plated in a 35-mm tissue culture plate. For transfection, 1.6 μg DNA (a mixture of CIBN-CaaX and ΔSV40-CRY2PHR-YFP-Raf1 at 1∶1 ratio) was mixed with 4.8 μL Turbofect in 160 μL DMEM medium and incubated at room temperature for 20 min. For neurite-outgrowth assay, 2.0×10^5^ PC12 cells were plated in a 12-well culture plate. For transfection, 1.2 μg DNA (a mixture of CIBN-GFP-CaaX and CRY2PHR-mCherry-Raf1 at 1∶1 ratio) was mixed with 3.6 μL Turbofect in 120 μL DMEM medium and added to each well. The DNA/Turbofect mixture was added to the cell culture drop-wise and incubated for 4 h before replenished with 2 mL standard culture medium.

### Western blot

For light-induced ERK activation (Fig. 2B–C) and p-ERK deactivation in darkness (Fig. 2D–E), 2.0×10^5^ NIH 3T3 fibroblast cells were plated in a 35-mm tissue culture plate and transfected with CIBN-CaaX and ΔSV40-CRY2PHR-YFP-Raf1. After recovering overnight from transfection, cells were serum starved in 0.1% FBS for another 24 h in order to reduce the base level of pErk. A mercury lamp was used as the light source. The cell culture was exposed to pulsed blue light filtered through a 472±30 nm bandpass filter (100-ms exposure time, 1-min interval, 2 mW/cm^2^ at the cell level). For light-induced ERK activation, four cultures were separately exposed to blue light for 0, 10, 20, and 30 min; for p-ERK deactivation, four cultures were first exposed to blue light for 10 min, and then incubated in dark for 0, 5, 10, and 30 min. A culture not exposed to light was used as the negative control. After light/dark treatment, cells were immediately washed and collected in ice-cold Dulbecco's phosphate-buffered saline (Cellgro). The cell lysates were separated by gel electrophoresis. Protein bands were transferred to a polyvinylidene fluoride membrane (Perkin Elmer) and probed with anti-pErk or anti-Erk antibody (9101S and 9102S, Cell Signaling Technology). HRP-conjugated secondary antibody was used for protein band detection.

For measurement of the activation of stress-activated protein kinase/c-Jun N-terminal kinase (SAPK/JNK) by blue and UV light (Fig. S1), 2.0×10^5^ NIH 3T3 fibroblast cells were plated in a 35-mm tissue culture plate and transfected with CIBN-CaaX and ΔSV40-CRY2PHR-YFP-Raf1. After recovering overnight from transfection, cells were serum starved in 0.1% FBS for another 24 h before exposed to light. Blue light stimulation was done by a train of 30-min pulses (2 mW/cm^2^, 100-ms exposure time, 1-min interval). UV light illumination (1 min duration, 302 nm, 1 mW/cm^2^) was followed by 30 min recovery in the complete medium in the 37°C incubator supplemented with 5% CO_2_. A cell culture plate incubated in dark was used as a negative control. After light treatment, cells were prepared for western blot analysis following the same protocol as mentioned above. Antibody against phosphorylated stress-activated protein kinase (p-SAPK/JNK, #4668, Cell Signaling Technology) was used to probe the photo-induced stress. Antibody against α/β tubulin (#2148, Cell Signaling Technology) was used to as a loading control.

### AKT Immunofluorescence staining

For immunofluorescence staining, 5.0×10^4^ PC12 cells were plated on a 12-mm PLL-coated round coverslip held in a 24-well plate and transfected with CIBN-GFP-CaaX and CRY2PHR-mCherry-Raf1. After 24-h recovery in full growth medium, the cell culture was washed and replaced with F12K medium without any serum. The cell cultures were then treated with either continuous blue light from the LED array (0.2 mW/cm^2^) for 24 h or kept in dark (negative control) in the same medium before fixation. As a positive control, starved cell culture was treated with 25% horse serum and 2.5% FBS for 5 min before fixation. All cell cultures were fixed with 4% formaldehyde diluted in warm PBS for 15 min at room temperature. After fixation, the fixative was removed and the cell cultures were rinsed three times in PBS buffer for 5 min each. The cell cultures were then blocked with 5% goat serum in PBS supplemented with 0.3% Triton-X 100 for 1 h at room temperature. The blocking buffer was then removed and the cell cultures were incubated with diluted primary antibody (1∶200 in PBS/1%BSA/0.3% Triton-X 100) for phosphorylated Akt (Cat. # 4060S, Cell Signaling Technology) overnight at 4°C. The cell cultures were rinsed with PBS 3 times and incubated with diluted (1∶1000 in PBS/1%BSA/0.3% Triton-X 100) Alexa Fluor 647-conjugated secondary antibody (#4414, Cell Signaling Technology) for 1.5 h at room temperature in the dark. After rinsing three times with PBS, antifade reagent was applied to each coverslip before fluorescence imaging. An oil-immersion 100× objective was used for cell imaging. Fluorescence from Alexa Fluor 647-conjugated secondary antibody was detected using the commercial Cy5 filter cube (Leica, excitation filter 620/60, dichroic mirror 660, emission filter 700/75).

### Live cell imaging

For the light-induced membrane recruitment assay, we used NIH 3T3 cells co-transfected with CIBN-GFP-CaaX and CRY2PHR-mCherry-Raf1 (or CRY2PHR-mCherry). Fluorescence imaging of the transfected cells were carried out on a confocal microscope (Nikon A1 confocal system) equipped with multiple laser lines and an oil-immersion 60× objective (Plan Apo VC, N.A. 1.4). The blue 488-nm laser line (0.5 mW, ∼2×10^6^ W/cm^2^ at the sample plane) of the confocal microscope was used as the stimulation light. The yellow 561-nm laser line was used to excite mCherry fluorescence without stimulating the CRY2PHR-CIBN interaction. A complete scanning of 512×512 pixels took 80 ms and the 488-nm stimulation scan was applied every 10 s followed by acquiring the mCherry fluorescence image using the 561-nm laser line.

For the ERK nuclear translocation assay, we used NIH 3T3 cells triply transfected with CIBN-CaaX, CRY2PHR-mCherry-Raf1, and ERK2-GFP. Transfected cells were serum-starved for 24 h before stimulation and imaging experiment. In addition to using the confocal microscope as mentioned above, we also used an epi-fluorescence microscope (Leica DMI6000B microscope) equipped with an oil-immersion 100× objective (HCXPL FLUOTAR, N.A. 1.3) and a mercury lamp as the light source. The power density for stimulating light (∼472 nm) was adjusted to ∼1 W/cm^2^ at the sample plane. Pulsed light (100-ms pulse duration, 30-s interval) was used for stimulation and imaging. The excitation light for the mCherry fluorescence (∼560 nm) does not activate CRY2PHR-CIBN binding. For fluorescence imaging in the immunofluorescence staining, fluorescence from GFP was detected using the commercial GFP filter cube (Leica, excitation filter 472/30, dichroic mirror 495, emission filter 520/35); fluorescence from mCherry was detected using the commercial Texas Red filter cube (Leica, excitation filter 560/40, dichroic mirror 595, emission filter 645/75).

### Construction of a programmable LED device

For long-term light illumination, a 4-by-3 blue LED array was constructed by assembling 12 blue LEDs (B4304H96, Linrose Electronics) on a breadboard. The LED device was controlled by a LabVIEW program through a data acquisition board (National Instrument-DAQ, PCI-6035E). The LEDs were supplied with user-defined DC voltages that were individually controllable through the LabVIEW program. The light intensity of each LED was further controlled through a tunable resistor. The breadboard was hosted in an aluminum box and a light diffuser film was positioned above the LED array to make the light intensity homogeneous in the defined area. To avoid cross illumination of different wells, separating barriers were placed around each LED. The light intensity at the cell culture plate was measured by a power meter (Newark, 1931-C).

### Long-term light stimulation for neurite outgrowth assay

PC12 cells were plated and transfected in a 12-well tissue culture plate. After transfection, cells were allowed to recover in high-serum F12K medium (15% horse serum +2.5% FBS) for 24 h. After recovery, the cell culture was exchanged to a low-serum medium (1.5% horse serum +0.25% FBS) to minimize the base-level ERK activation by serum. For light stimulation, the 12-well plate was placed onto the LED device with each well aligned above each LED. By adjusting the DC voltage and the resistors, the light intensity was tunable in the range of 0.025–5 mW/cm^2^ at the cell level. Both the LED device and the cell culture plate were placed into a CO_2_ incubator. Each well was stimulated by one LED and 12 cultures were simultaneously illuminated in one batch of experiment. Neurite outgrowth was quantified after 24 h, 36 h or 72 h of light stimulation.

### Determination of the expression level of CRY2PHR-mCherry-Raf1

We used the same excitation light power and excitation filter/dichroic mirror/emission filter for all imaging experiments in the neurite outgrowth assay. Fluorescence of GFP was detected using the GFP filter cube (Leica, excitation filter 472/30, dichroic mirror 495, emission filter 520/35); fluorescence from mCherry was detected using the commercial Texas Red filter cube (Leica, excitation filter 560/40, dichroic mirror 595, emission filter 645/75). The relative CRY2PHR-mCherry-Raf1 expression level was estimated by the mCherry fluorescence intensity. ImageJ was used to measure the average intensity of a transfected cell and that of the background. The CRY2PHR-mCherry-Raf1 expression level was thus proportional to the background-subtracted fluorescence intensity. We then sorted the intensity into three levels referred to as low, medium, and high corresponding to 0-30%, 31-75%, and 75-100% of the maximal background-subtracted fluorescence intensity (Fig. S7).

### Kinetic analysis

For kinetic analysis of membrane recruitment assay, the percentage of membrane-associated CRY2PHR-mCherry-Raf1 (or CRY2PHR-mCherry) was calculated by dividing the average fluorescence intensity (per pixel) of the membrane by that of the membrane and the cytoplasm I_mem_/(I_mem_ + I_cyto_). The time-dependent change of the percentage was normalized between 0 and 1 for each cell and then averaged over 8 (CRY2PHR-mCherry) and 4 (CRY2PHR-mCherry-Raf1) cells to obtain the kinetic plots in Fig. 1C-D. For kinetic analysis of ERK nuclear translocation assay, the ratio of nuclear/cytoplasmic ERK2-GFP was calculated by dividing the average fluorescence intensity in the cell nucleus by that of the cytoplasm (I_nucl_/I_cyto_). The time-dependent change of the ratio was normalized between 0 and 1 for each cell and then averaged over 10 cells to obtain the kinetic plots in Fig. 3C.

### Neurite length measurement

For non-transfected cells, bright-field images were used for neurite length measurement. For transfected cells, fluorescence images were used to identify singly transfected or co-transfected cells. For each cell, the longest neurite was selected and its length measured from the perinuclear region to the neurite terminus using the NeuronJ plugin of ImageJ software [63].

### Statistical analysis

The p-values were determined by performing two-tailed, unpaired t-test using the GraphPad Prism software.

## Supporting Information

Figure S1 **Phototoxicity calibration for light stimulation.** Western blot analysis showed that blue light stimulation induced negligible activation of stress-activated protein kinase pathway. NIH3T3 cells were exposed 30-min blue light stimulation (488 nm, 2 mW/cm^2^, 100-ms exposure time, 1-min interval), the same condition used to probe ERK activity by Western blot (Fig. 2B-C in the main text). Blotting by antibodies against phosphorylated SAPK/JNK revealed negligible activation of stress-activated protein kinase. As a positive control, NIH3T3 cells were exposed to 1-min UV light (302 nm, 1 mW/cm^2^). As expected, 1-min UV exposure led to significant activation of p-SAPK/JNK. Antibody against α/β tubulin was used as a loading control.
(TIF)

Figure S2 **The kinetics of ERK2-GFP nuclear translocation can be tuned by varying the frequency of stimulating light.** A train of 30-s interval light pulses (200 ms duration per pulse) resulted in maximum nuclear ERK2-GFP fluorescence levels around 15 min. This activation time decreased to 5 min when the pulse interval was reduced to 2 s (200 ms duration per pulse). Data were averaged over 10 cells and were presented by mean ± standard error (s.e.m.).
(TIF)

Figure S3 **Comparison of neurite outgrowth in PC12 cells under different conditions.** Cells co-transfected with CIBN-GFP-CaaX and CRY2PHR-mCherry-Raf1 grew significantly longer neurites under light stimulation compared to those in dark (A). Cells singly transfected with either CRY2PHR-mCherry-Raf1 (B), singly transfected with CIBN-GFP-CaaX (C), or co-transfected with CIBN-GFP-CaaX and CRY2PHR-mCherry (D) did not show marked neurite outgrowth. When treated with NGF, cells grew much longer neurites than those without NGF treatment, either with or without light stimulation (E).
(TIF)

Figure S4 **Effect of constitutive active and wild type Raf1 overexpression on PC12 neurite outgrowth.** PC12 cells were transfected with (A) Raf1-GFP-CaaX (a membrane-anchored constitutive active form) and (B) Raf1-GFP (wild type) and incubated in starvation medium for 3 days. Significant neurite outgrowth can only be observed by cells transfected with Raf1-GFP-CaaX but not Raf1-GFP.
(TIF)

Figure S5 **Effect of inhibitors on NGF-induced neurite outgrowth.** Both K252A (TrkA inhibitor) and U0126 (MEK inhibitor) completely blocked the NGF-induced neurite outgrowth in CIBN-GFP-CaaX and CRY2PHR-mCherry-Raf1 co-transfected cells.
(TIF)

Figure S6 **Representative images of light-induced neurite outgrowth with larger field of view.** (A) Light-induced neurite outgrowth for cells transfected with CIBN-GFP-CaaX and CRY2PHR-mCherry-wtRaf1 under blue light. (B) Snapshot of traces of longest neurite generated by the ImageJ plugin NeuronJ. (C) Neurite outgrowth for cells transfected with CIBN-GFP-CaaX and CRY2PHR-mCherry-wtRaf1 in dark. (D) Neurite outgrowth for cells transfected with CIBN-GFP-CaaX and CRY2PHR-mCherry-dnRaf1 under blue light. (E) Neurite outgrowth for cells transfected with CIBN-GFP-CaaX and CRY2PHR-mCherry-wtRaf1 under blue light with the TrkA inhibitor K252A (E) or the MEK inhibitor U0126 (F).
(TIF)

Figure S7 **Dependence of cell morphology on the level of CRY2PHR-mCherry-Raf1 expression.** (A) The average neurite length by NGF stimulation (white bars) remained constant for low, medium, and high levels of CRY2PHR-mCherry-Raf1 expression. The average neurite length by light stimulation (gray bars) showed slightly larger fluctuation, possibly due to the more polarized cell morphology induced by the light-activated Raf/MEK/ERK signaling pathway (see Fig. 5 in the main text). (B) The average neurite number per cell remained constant for both NGF (white bars) and light (gray bars) stimulation at various levels of CRY2PHR-mCherry-Raf1 expression.
(TIF)

Figure S8 **Scatter plots of light-induced neurite outgrowth under different temporal stimulation.** (A–C) The average neurite lengths for 15-min (A), 45-min (B), and 75-min (C) on-time per cycle with different off-time. In all three cases, when the off-time was less than 45 min, the average neurite length was comparable to that induced by continuous light stimulation. When the off-time was beyond 45 min, both the average length and the distribution span decreased. (D) For 45-min off-time with different on-time, when the on-time was equal to or longer than 5 min, the average neurite length was comparable to that induced by continuous light stimulation. A 1-min on-time induced shorter neurite length with decreased distribution span as well. These results showed that as the cumulative activation time of the Raf/MEK/ERK decreased, the whole co-transfected cell population displayed shorter neurites.
(TIF)

Figure S9 **Absolute neurite length from two independent sets of experiments of light-induced neurite outgrowth vs. the light intensity.** PC12 cells co-transfected with CIBN-GFP-CaaX and CRY2PHR-mCherry-Raf1 were exposed to blue light with different intensity for 24 h. Results showed the same dependence of the neurite length on the light intensity.
(TIF)

Figure S10 **Results of two independent sets of experiments of light-induced neurite outgrowth with different on-time.** PC12 cells were co-transfected with CIBN-GFP-CaaX and CRY2PHR-mCherry-Raf1 and were exposed to blue light at 0.2 mW/cm^2^ for 36 h. (A–B) Absolute neurite lengths from two sets of 45-min on-time and various off-time experiments. Batch-to-batch variation in neurite length was ∼15%. Within each set, the 45-min off-time threshold was repeated. (C–E) Overlaid normalized neurite lengths from two sets of experiment with 15-min (C), 45-min (D), and 75-min (E) on-time and various off-time. Within each set of experiment, the 45-min off-time threshold was repeated. (F) Overlaid normalized neurite length from two sets of experiments with 45-min off time and various on-times. Within each set, 1-min on-time induced significantly shorter neurite length than 5, 10, 15, 45, and 75 min on-time did.
(TIF)

Table S1 **Sequences of DNA oligonucleotides used in this study.**
(DOC)

Table S2 **Protocols of the 2-step overlap extension PCR used in this study.**
(DOC)

Movie S1 **Light-mediated membrane recruitment and dissociation of Raf1 in NIH3T3 cells co-transfected with CIBN-GFP-CaaX and CRY2PHR-mCherry-Raf1.** A Nikon A1 confocal microscope was used for image acquisition. The blue 488-nm laser line (0.5 mW, ∼2×10^6^ W/cm^2^ at the sample plane with a 60× objective) of the confocal microscope was used as the stimulation light. A complete scanning of each frame (512×512 pixels) took 80 ms and a stimulation was applied every 10 s followed by acquiring the mCherry fluorescence image.
(AVI)

Movie S2 **ERK2-GFP nuclear translocation induced by light-mediated membrane recruitment of Raf1.** Time-stamped images were acquired using the same microscope and protocol as those in Movie S1. The same field of view is shown in Fig. 2A.
(AVI)

Movie S3 **Light-induced membrane recruitment of Raf1 resulted in prolonged retainment of nuclear ERK2-GFP.** NIH3T3 cells were triply transfected with CIBN-CaaX, CRY2PHR-mCherry-Raf1, and ERK2-GFP. An epi-fluorescence microscope (Leica DMI6000B) equipped with an oil-immersion 100× objective was used for image acquisition. The blue light (∼472 nm) filtered from a mercury lamp was used as the stimulation light, whose power density was adjusted to ∼1 W/cm^2^ at the sample plane. Pulsed blue light (100-ms pulse duration, 30-s interval) was used for stimulation and imaging. After each pulse of blue light stimulation, a green light pulse was applied to acquire the fluorescence image of CRY2PHR-mCherry-Raf1. The same field of view is shown in Fig. 3A.
(AVI)

Movie S4 **EGF induced a transient nuclear translocation of ERK2-GFP.** NIH3T3 cells were transfected with ERK2-GFP. Time-stamped images were acquired using the same microscope and protocol as those in Movie S3 except that no green light pulse was applied after each blue light pulse. The same field of view is shown in Fig. 3B.
(AVI)

Movie S5 **Reversible light-controlled ERK2-GFP nuclear translocation.** NIH3T3 cells were triply transfected with CIBN-CaaX, CRY2PHR-mCherry-Raf1, and ERK2-GFP. Time-stamped images were acquired using the same microscope and protocol as those in Movie S3. The same field of view is shown in Fig. 3D.
(AVI)
