# Supplementary figures and images for: Light-Mediated Kinetic Control Reveals the Temporal Effect of the Raf/MEK/ERK Pathway in PC12 Cell Neurite Outgrowth
Source: PLoS One. 2014 Mar 25;9(3):e92917. doi: 10.1371/journal.pone.0092917 (PMC3965503; doi:10.1371/journal.pone.0092917)

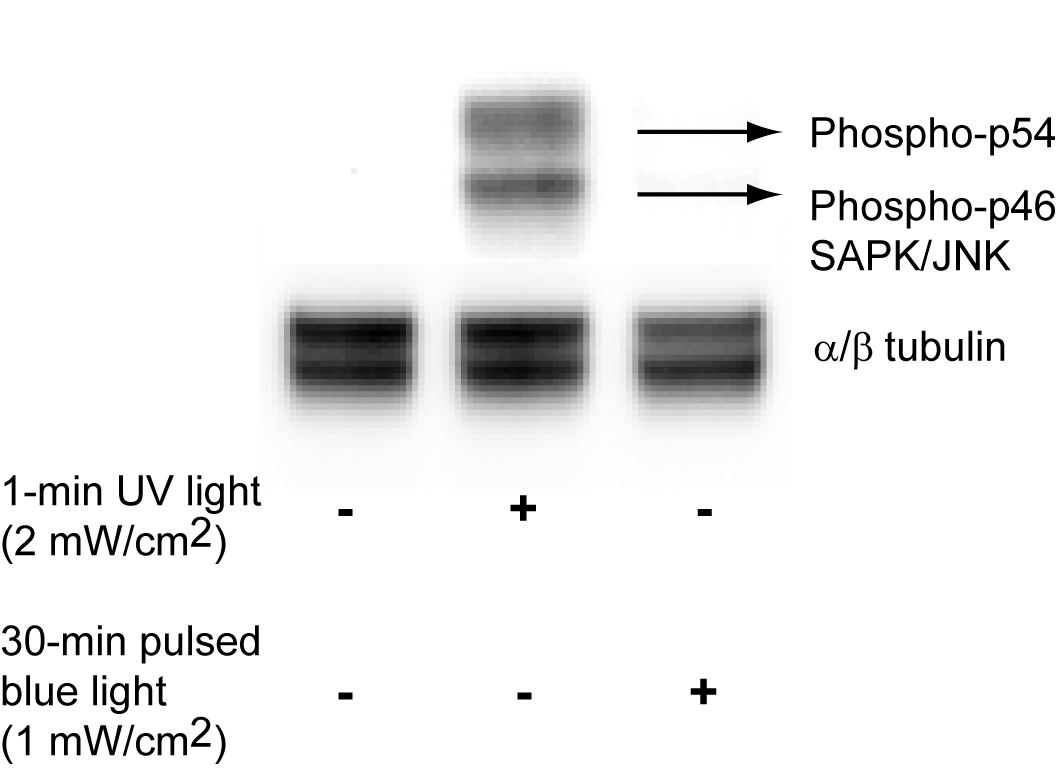

Supplement: Figure S1 — Phototoxicity calibration for light stimulation. Western blot analysis showed that blue light stimulation induced negligible activation of stress-activated protein kinase pathway. NIH3T3 cells were exposed 30-min blue light stimulation (488 nm, 2 mW/cm2, 100-ms exposure time, 1-min interval), the same condition used to probe ERK activity by Western blot (Fig. 2B-C in the main text). Blotting by antibodies against phosphorylated SAPK/JNK revealed negligible activation of stress-activated protein kinase. As a positive control, NIH3T3 cells were exposed to 1-min UV light (302 nm, 1 mW/cm2). As expected, 1-min UV exposure led to significant activation of p-SAPK/JNK. Antibody against α/β tubulin was used as a loading control. (TIF) [file pone.0092917.s001.tif]

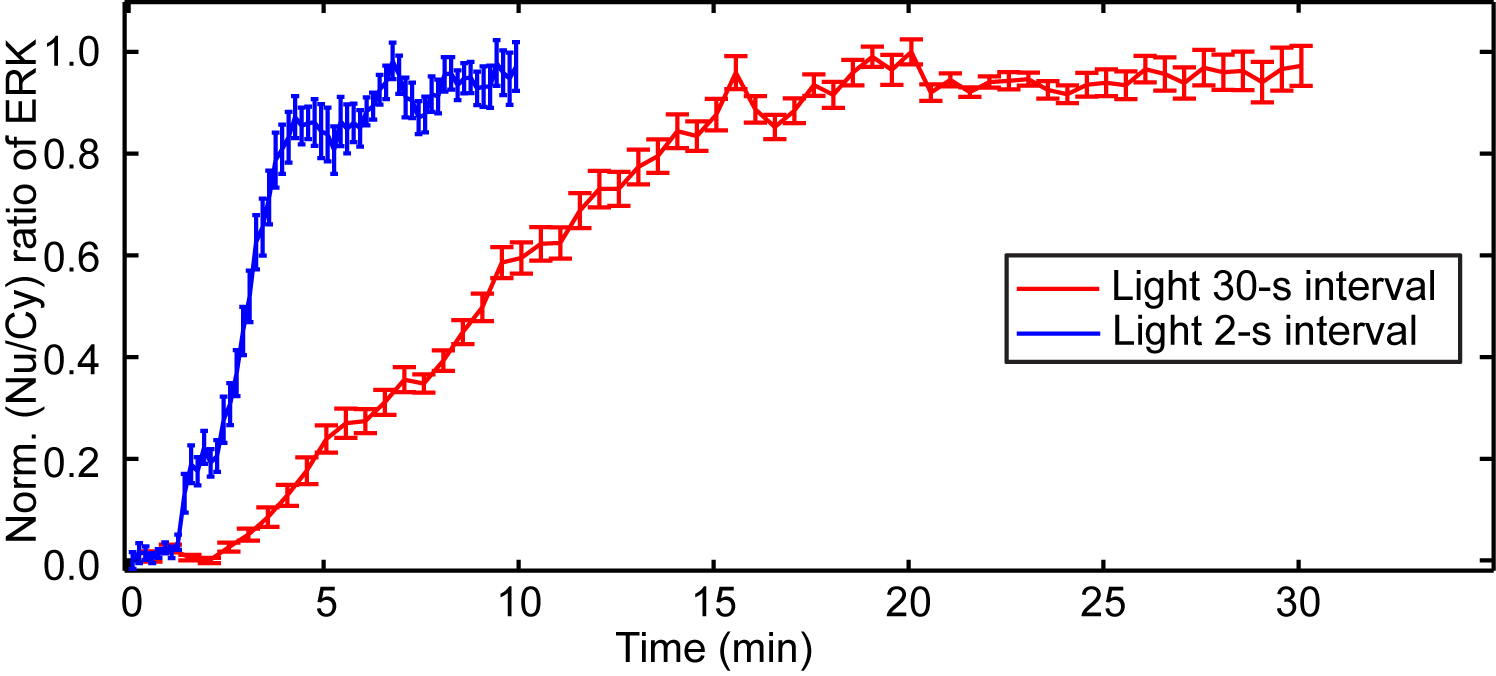

Supplement: Figure S2 — The kinetics of ERK2-GFP nuclear translocation can be tuned by varying the frequency of stimulating light. A train of 30-s interval light pulses (200 ms duration per pulse) resulted in maximum nuclear ERK2-GFP fluorescence levels around 15 min. This activation time decreased to 5 min when the pulse interval was reduced to 2 s (200 ms duration per pulse). Data were averaged over 10 cells and were presented by mean ± standard error (s.e.m.). (TIF) [file pone.0092917.s002.tif]

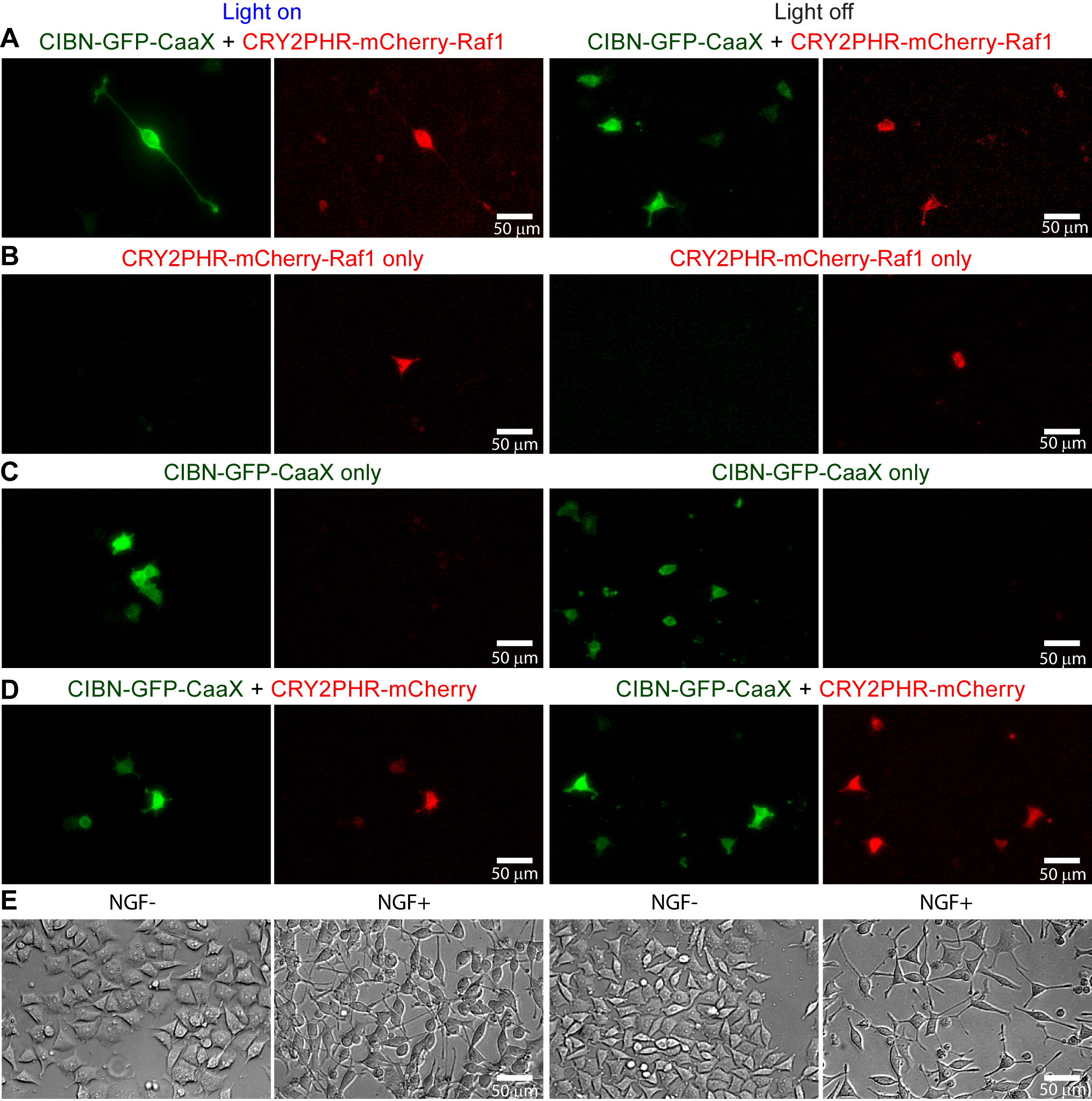

Supplement: Figure S3 — Comparison of neurite outgrowth in PC12 cells under different conditions. Cells co-transfected with CIBN-GFP-CaaX and CRY2PHR-mCherry-Raf1 grew significantly longer neurites under light stimulation compared to those in dark (A). Cells singly transfected with either CRY2PHR-mCherry-Raf1 (B), singly transfected with CIBN-GFP-CaaX (C), or co-transfected with CIBN-GFP-CaaX and CRY2PHR-mCherry (D) did not show marked neurite outgrowth. When treated with NGF, cells grew much longer neurites than those without NGF treatment, either with or without light stimulation (E). (TIF) [file pone.0092917.s003.tif]

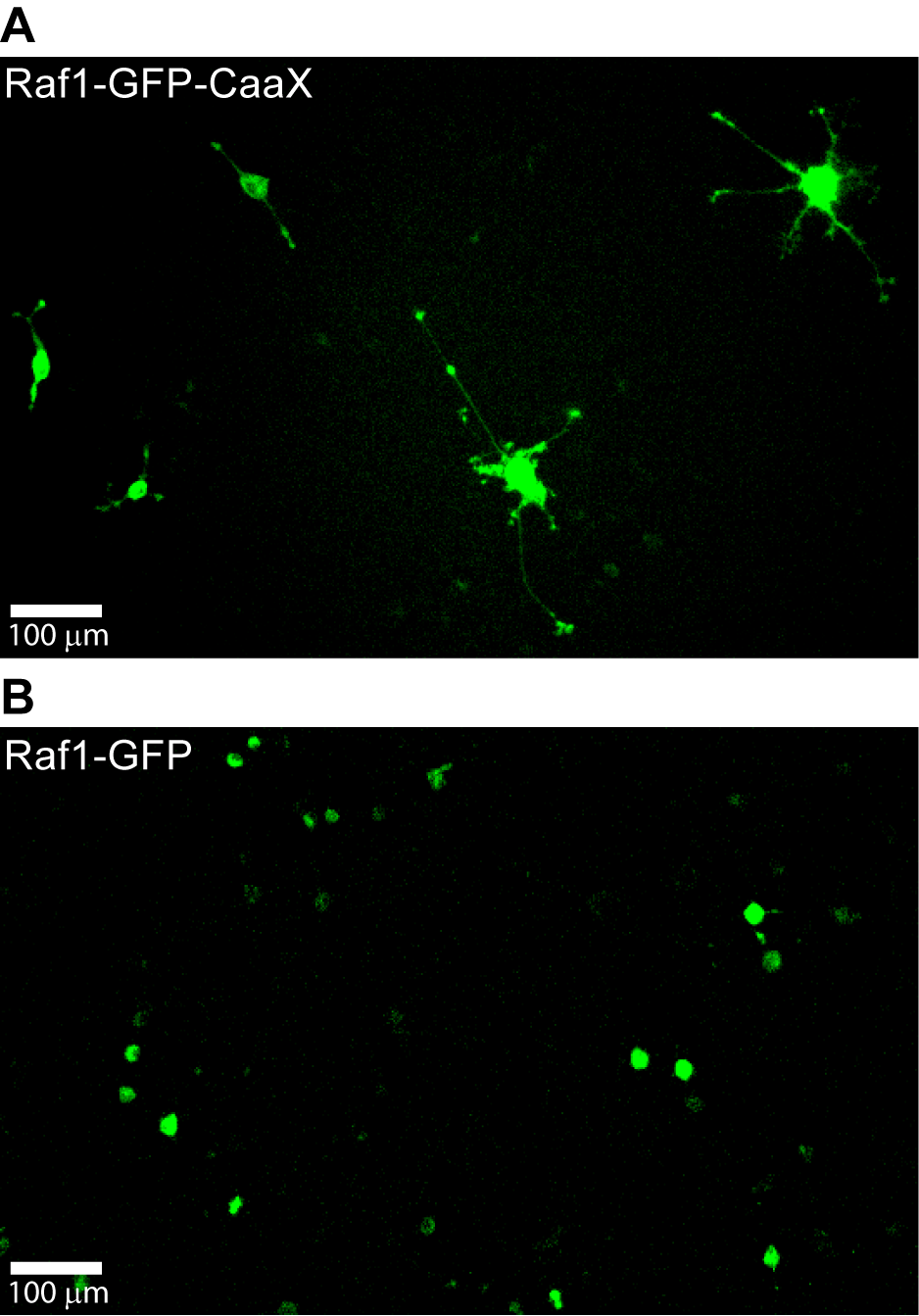

Supplement: Figure S4 — Effect of constitutive active and wild type Raf1 overexpression on PC12 neurite outgrowth. PC12 cells were transfected with (A) Raf1-GFP-CaaX (a membrane-anchored constitutive active form) and (B) Raf1-GFP (wild type) and incubated in starvation medium for 3 days. Significant neurite outgrowth can only be observed by cells transfected with Raf1-GFP-CaaX but not Raf1-GFP. (TIF) [file pone.0092917.s004.tif]

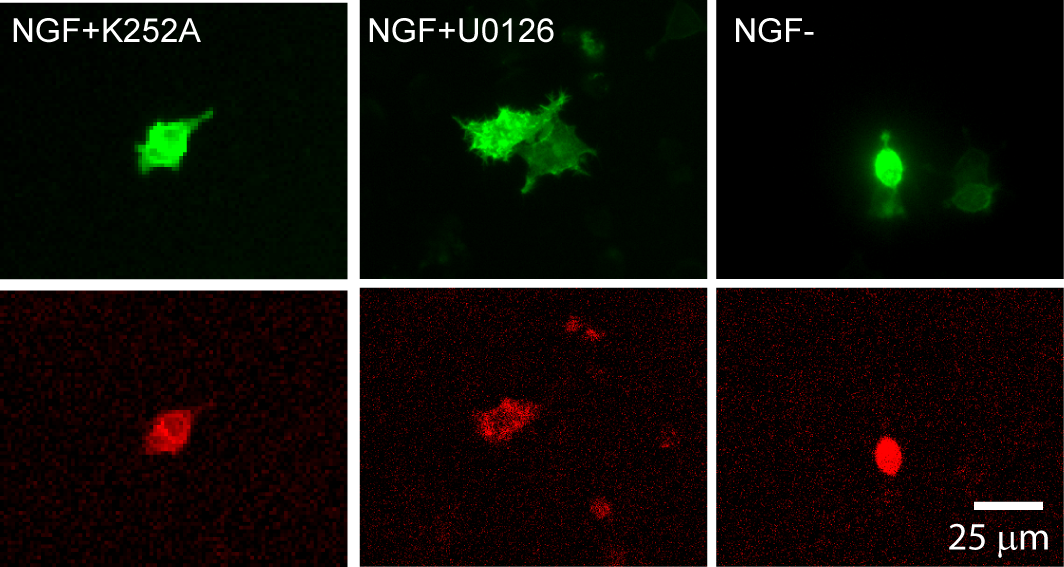

Supplement: Figure S5 — Effect of inhibitors on NGF-induced neurite outgrowth. Both K252A (TrkA inhibitor) and U0126 (MEK inhibitor) completely blocked the NGF-induced neurite outgrowth in CIBN-GFP-CaaX and CRY2PHR-mCherry-Raf1 co-transfected cells. (TIF) [file pone.0092917.s005.tif]

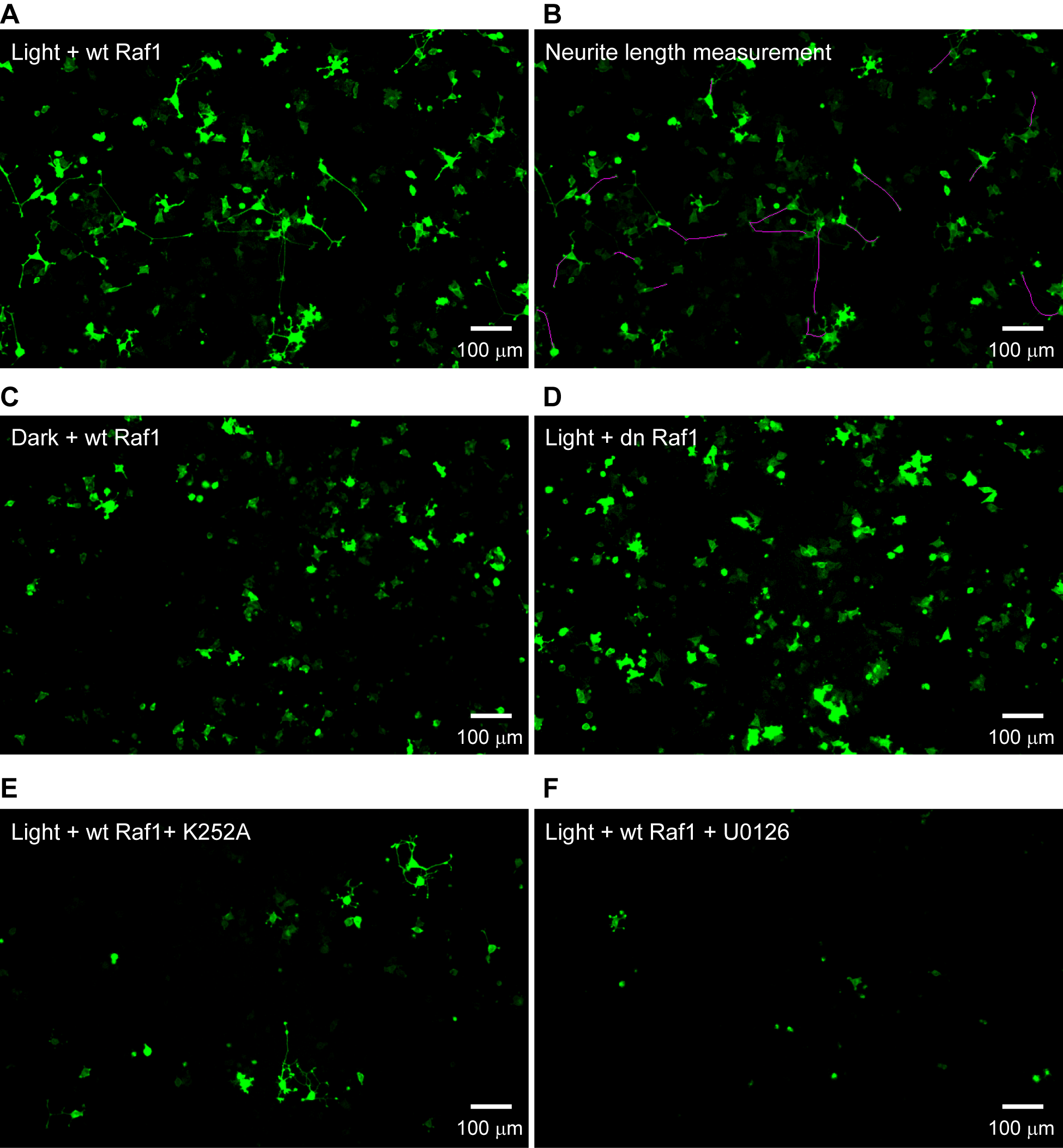

Supplement: Figure S6 — Representative images of light-induced neurite outgrowth with larger field of view. (A) Light-induced neurite outgrowth for cells transfected with CIBN-GFP-CaaX and CRY2PHR-mCherry-wtRaf1 under blue light. (B) Snapshot of traces of longest neurite generated by the ImageJ plugin NeuronJ. (C) Neurite outgrowth for cells transfected with CIBN-GFP-CaaX and CRY2PHR-mCherry-wtRaf1 in dark. (D) Neurite outgrowth for cells transfected with CIBN-GFP-CaaX and CRY2PHR-mCherry-dnRaf1 under blue light. (E) Neurite outgrowth for cells transfected with CIBN-GFP-CaaX and CRY2PHR-mCherry-wtRaf1 under blue light with the TrkA inhibitor K252A (E) or the MEK inhibitor U0126 (F). (TIF) [file pone.0092917.s006.tif]

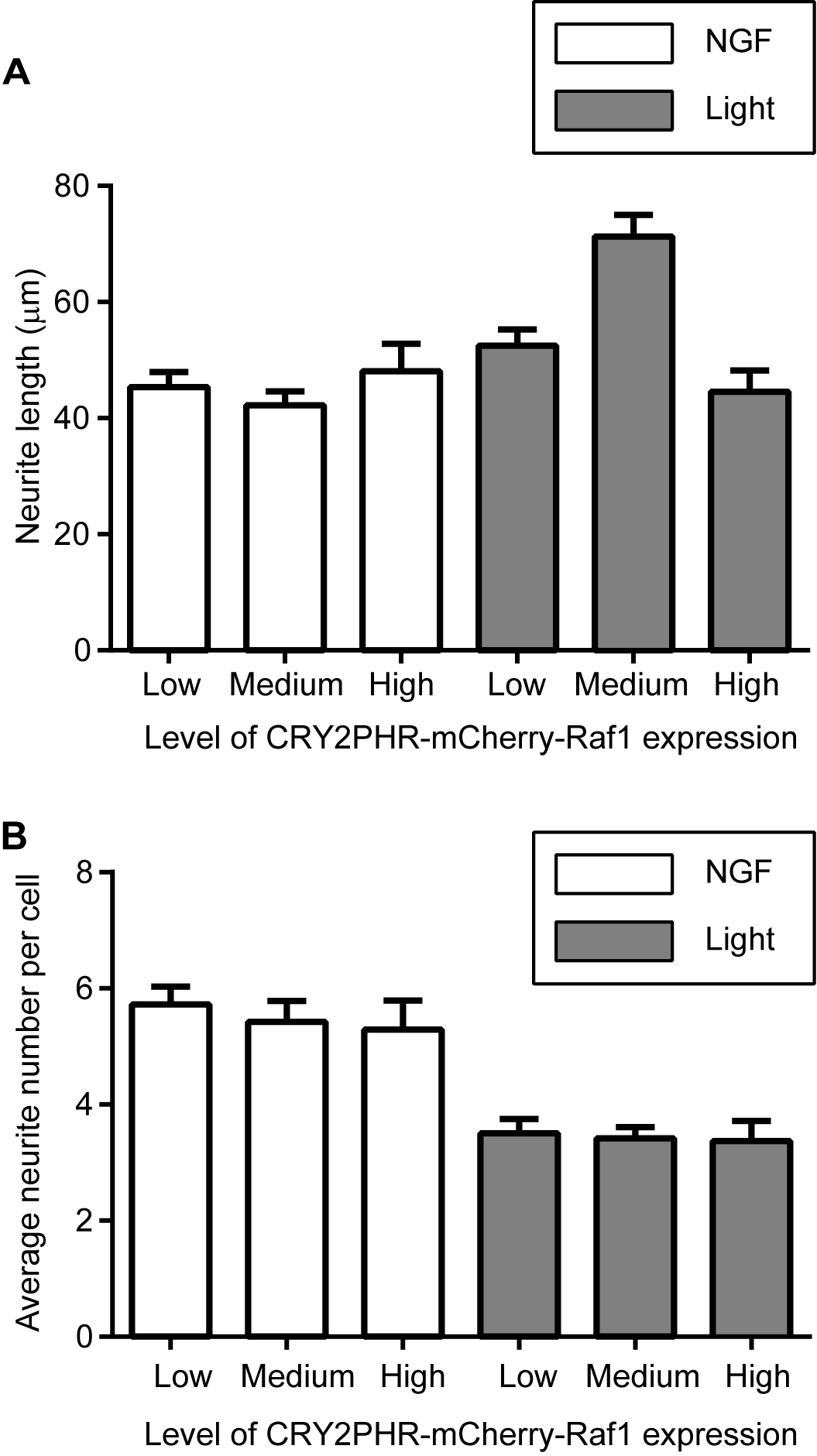

Supplement: Figure S7 — Dependence of cell morphology on the level of CRY2PHR-mCherry-Raf1 expression. (A) The average neurite length by NGF stimulation (white bars) remained constant for low, medium, and high levels of CRY2PHR-mCherry-Raf1 expression. The average neurite length by light stimulation (gray bars) showed slightly larger fluctuation, possibly due to the more polarized cell morphology induced by the light-activated Raf/MEK/ERK signaling pathway (see Fig. 5 in the main text). (B) The average neurite number per cell remained constant for both NGF (white bars) and light (gray bars) stimulation at various levels of CRY2PHR-mCherry-Raf1 expression. (TIF) [file pone.0092917.s007.tif]

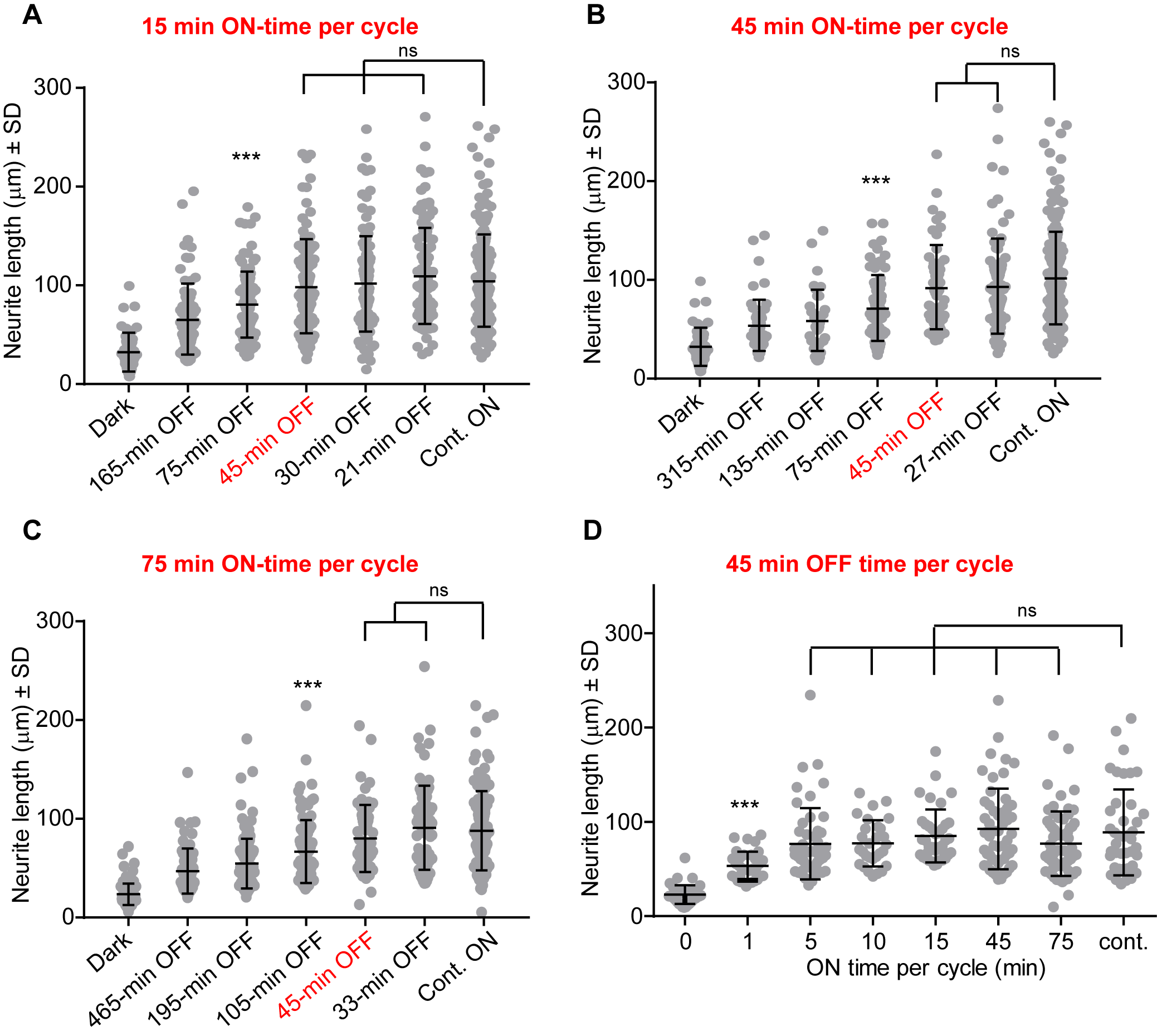

Supplement: Figure S8 — Scatter plots of light-induced neurite outgrowth under different temporal stimulation. (A–C) The average neurite lengths for 15-min (A), 45-min (B), and 75-min (C) on-time per cycle with different off-time. In all three cases, when the off-time was less than 45 min, the average neurite length was comparable to that induced by continuous light stimulation. When the off-time was beyond 45 min, both the average length and the distribution span decreased. (D) For 45-min off-time with different on-time, when the on-time was equal to or longer than 5 min, the average neurite length was comparable to that induced by continuous light stimulation. A 1-min on-time induced shorter neurite length with decreased distribution span as well. These results showed that as the cumulative activation time of the Raf/MEK/ERK decreased, the whole co-transfected cell population displayed shorter neurites. (TIF) [file pone.0092917.s008.tif]

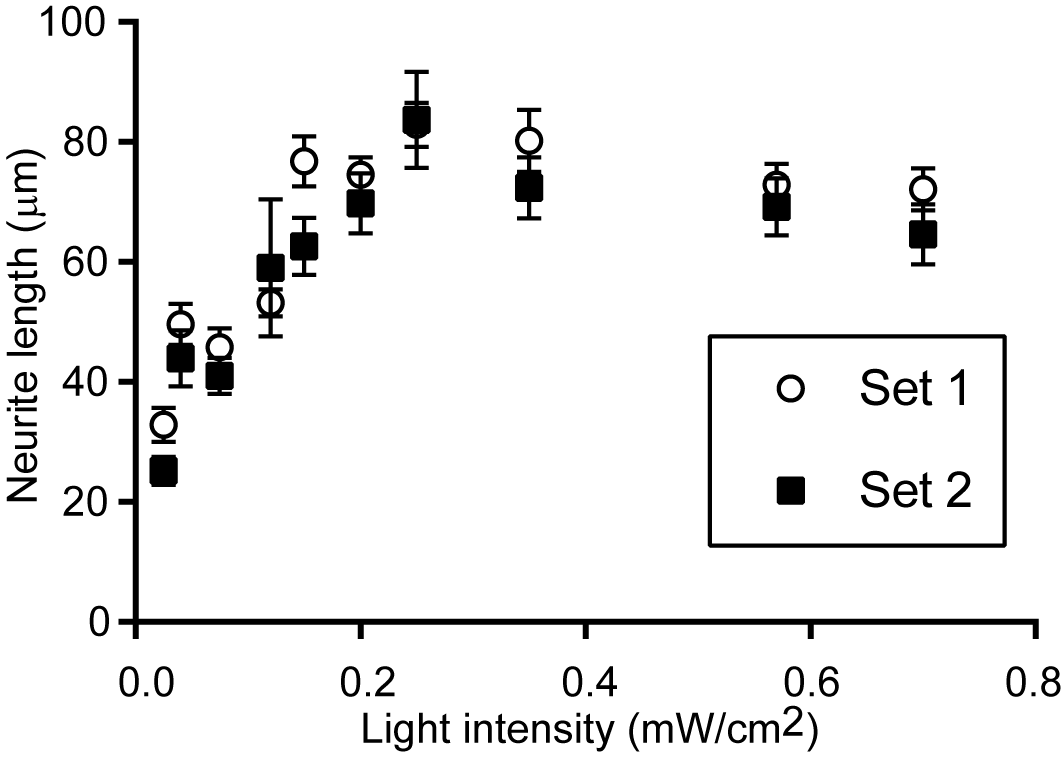

Supplement: Figure S9 — Absolute neurite length from two independent sets of experiments of light-induced neurite outgrowth vs. the light intensity. PC12 cells co-transfected with CIBN-GFP-CaaX and CRY2PHR-mCherry-Raf1 were exposed to blue light with different intensity for 24 h. Results showed the same dependence of the neurite length on the light intensity. (TIF) [file pone.0092917.s009.tif]

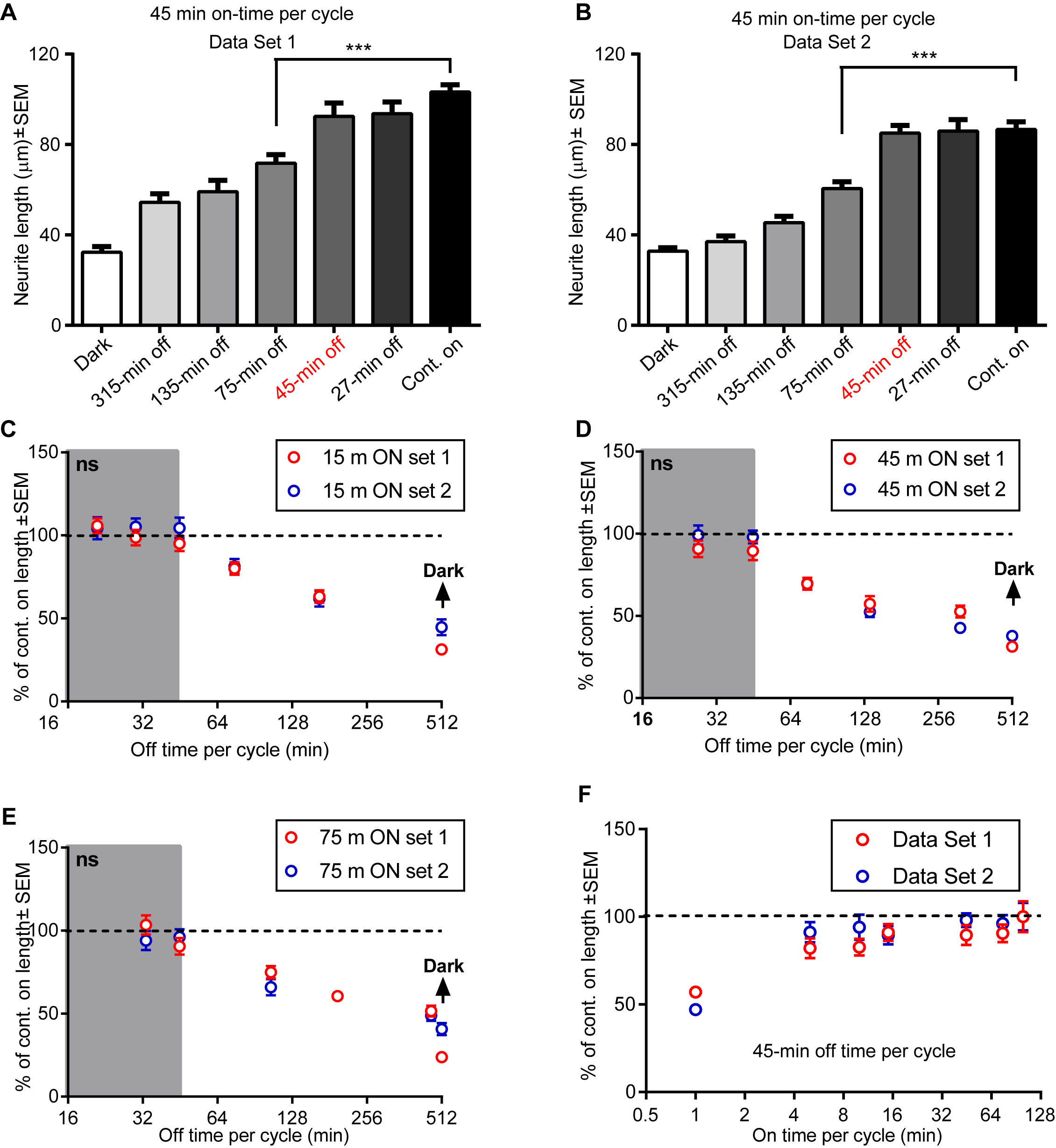

Supplement: Figure S10 — Results of two independent sets of experiments of light-induced neurite outgrowth with different on-time. PC12 cells were co-transfected with CIBN-GFP-CaaX and CRY2PHR-mCherry-Raf1 and were exposed to blue light at 0.2 mW/cm2 for 36 h. (A–B) Absolute neurite lengths from two sets of 45-min on-time and various off-time experiments. Batch-to-batch variation in neurite length was ∼15%. Within each set, the 45-min off-time threshold was repeated. (C–E) Overlaid normalized neurite lengths from two sets of experiment with 15-min (C), 45-min (D), and 75-min (E) on-time and various off-time. Within each set of experiment, the 45-min off-time threshold was repeated. (F) Overlaid normalized neurite length from two sets of experiments with 45-min off time and various on-times. Within each set, 1-min on-time induced significantly shorter neurite length than 5, 10, 15, 45, and 75 min on-time did. (TIF) [file pone.0092917.s010.tif]
