# Supplementary material for: Light-Mediated Kinetic Control Reveals the Temporal Effect of the Raf/MEK/ERK Pathway in PC12 Cell Neurite Outgrowth
Source: PLoS One. 2014 Mar 25;9(3):e92917. doi: 10.1371/journal.pone.0092917 (PMC3965503; doi:10.1371/journal.pone.0092917)
Supplement: Table S1 — Sequences of DNA oligonucleotides used in this study. (DOC) [file pone.0092917.s011.doc]

**Table S1**.

| **Plasmid** | **PCR segment** | **Template** | **Sense primer** | **Antisense primer** |
| --- | --- | --- | --- | --- |
| **CIBN-CaaX** | None | CIBN-GFP-CaaX | acggattatattcatgtaccggtcgccaccggtaaaaagaagaaaaagaagtcaaagaca | gagctcgagatctgagtccggacttgtacactacataattacacactttgtctttgactt |
| **CRY2PHR-mCherry-Raf1** | Raf1 | CRY2PHR-mCherry-Tubulin | ctgtacaagtccggactcagatctcgagtgatggagcacatacagggagcttggaagacg | acgggccctctagactcgagcggccgcttagaagacaggcagcctcggggacgtggtcag |
| **CRY2PHR-YFP-Raf1** | YFP | CRY2PHR-mCherry-Raf1 | gctggtagtgctggtagtgctggtactagtatggtgagcaagggcgaggagctgttcacc | gtgctccatcactcgagatctgagtccggacttgtacagctcgtccatgccgagagtgat |
| **ΔSV40-CRY2PHR-YFP-Raf1** | ΔSV40 | CMV-CRY2PHR-YFP-Raf1 | tacaacaaggcaaggcttgaccgacaattgaggcaggcagaagtatgcaaagcatgc | ctttttgtccatcttcatggtaccaagcttagcctaggcctccaaaaaagcctcctcact |
