# Supplementary material for: Light-Mediated Kinetic Control Reveals the Temporal Effect of the Raf/MEK/ERK Pathway in PC12 Cell Neurite Outgrowth
Source: PLoS One. 2014 Mar 25;9(3):e92917. doi: 10.1371/journal.pone.0092917 (PMC3965503; doi:10.1371/journal.pone.0092917)
Supplement: Table S2 — Protocols of the 2-step overlap extension PCR used in this study. (DOC) [file pone.0092917.s012.doc]

**Table S2**.

| **First step** | | | **Second step** | | |
| --- | --- | --- | --- | --- | --- |
| **Material** | **Conc.** | **Vol. ( µL)** | **Material** | **Conc.** | **Vol. ( µL)** |
| H_2_O |  | 35.5 | H_2_O |  | 4.4 |
| Buffer | 5× | 10 | Buffer | 5× | 4 |
| dNTP | 10 mM | 1 | dNTP | 10 mM | 0.4 |
| Sense primer | 25 µM | 1 | PCR segment | 75 ng/uL | 10 |
| Antisense primer | 25 µM | 1 |  |  |  |
| Template | 10 ng/uL | 1 | Template | 3 ng/uL | 1 |
| Polymerase | 2 U/uL | 0.5 | Polymerase | 2 U/uL | 0.2 |
| Total volume |  | 50 | Total **v**olume |  | 20 |
|  |  |  |  |  |  |
| **PCR program** | | | **PCR program** | | |
| **Step** | **Temperature** | **Time** | **Step** | **Temperature** | **Time** |
| **1** | 98 °C | 3 min | 1 | 98 °C | 3 min |
| **2** | 98 °C | 30 s | 2 | 98 °C | 30 s |
| **3** | 69 °C | 30 s | 3 | 55 °C | 30 s |
| **4** | 72 °C | 23 s | 4 | 72 °C | 15 min |
| **5** | GOTO 2 | Rep. 35 | 5 | GOTO 2 | Rep. 18 |
| **6** | 72 °C | 10 min | 6 | 72 °C | 10 min |
| **7** | Hold | 4 °C | **7** | Hold | 4 °C |
